# T2T-CHM13 improves read mapping and detection of clinically relevant genetic variation in the Swedish population

**DOI:** 10.1101/gr.279320.124

**Published:** 2025-11

**Authors:** Daniel Schmitz, Adam Ameur, Åsa Johansson

**Affiliations:** Department of Immunology, Genetics, and Pathology, Science for Life Laboratory, Uppsala University, 751 08 Uppsala, Sweden

## Abstract

The T2T-CHM13 reference genome, released in March 2022, fills in the 8% of the human genome that was not resolved in GRCh38 and reconstructs large parts of the known genome. The more accurate and complete reference genome is expected to improve the quality of read mapping and variant calling. Even though whole-genome sequencing (WGS)-based approaches have become the gold standard in medical genetics, the extent of the benefits of the improved reference genome remains unclear. In this study, we aim to evaluate alignment and variant call performance with T2T-CHM13 as a reference using a cross-sectional Swedish cohort (SweGen) comprising 1000 individuals with short-read Illumina WGS data available. Remapping and variant calling is performed using the nf-core/sarek pipeline. T2T-CHM13 improves a wide range of mapping- and variant calling-related metrics, including a higher fraction of properly paired reads, lower mismatch rate, and more uniform coverage of coding regions. Moreover, the fraction of ambiguous alignments is higher, reflecting segmental duplications that were incorrectly collapsed in GRCh37 and GRCh38. In comparison to GRCh38, we identify 10 million additional variants in the cohort, including 5.5 million singletons, and observe an increased sensitivity for rare variants. SnpEff assigns impact ratings of moderate or high to 13% more variants in T2T-CHM13 than GRCh38. In summary, we conclude that T2T-CHM13 improves alignment metrics with higher alignment quality, better variant calling performance, and confidence, including for rare and deleterious variants. The T2T-CHM13 genome reference thus facilitates enhanced discovery of new disease-causing variation, benefiting, for example, rare-disease diagnostics.

The first draft of the human reference genome was published by the Human Genome Sequencing Consortium in 2001 (International Human Genome Sequencing Consortium 2001). Since then, the reference genome has been continuously improved with patch releases, which add missing sequences, correct assembly errors, and augment the genome with alternative haplotypes. In 2022, the Telomere-to-Telomere (T2T) Consortium published the first gapless T2T assembly of a human genome (Nurk et al. 2022), and in 2023, an update which includes Chromosome Y (Rhie et al. 2023). This assembly fills in more than 200 Mbp of missing sequences from GRCh38, which corresponds to ∼8% of the human genome. Among the regions that are now resolved are centromeres, the short arms of the acrocentric Chromosomes 13, 14, 15, 21, and 22, as well as the majority of Chromosome Y. In addition to resolving these unknown regions, they reported novel findings about the structure of the human genome. The T2T Consortium discovered 3604 new genes and identified around 50 Mbp of segmental duplications (SDs), which make up half of all resolved gaps. Overall, SDs are a common genomic feature, representing 7% of the whole human genome, which are especially common on the short arms of acrocentric chromosomes (Vollger et al. 2022). Moreover, the T2T consortium benchmarked their assembly's performance for variant calling and found it to allow for the detection of many novel variants, especially in the previously unresolved regions. They also found that their novel reference reduces the number of false-positive calls mainly in protein-coding genes, and they highlight the increased sensitivity for detection of rare variants and singletons (Aganezov et al. 2022). The T2T-CHM13 genome has already seen use in the evaluation of novel tools (Formenti et al. 2022; Mc Cartney et al. 2022), characterization of SVs (Prodanov and Bansal 2022), and epigenetics (Gershman et al. 2022).

During recent years, whole-genome sequencing (WGS) has been introduced as a clinical diagnostic tool, especially for rare diseases, which affect between 3.5% and 5.9% of the population (Nguengang Wakap et al. 2020) and are often caused by a single nucleotide variant (SNV) or a structural variant (SV). However, a minority of the rare disease patients receive a definite diagnosis and often must go through a so-called “diagnostic odyssey” of repeated visits to specialists before a conclusive result can be obtained (Molster et al. 2016; Yan et al. 2020). A recent meta-analysis found WGS to significantly improve diagnostic yield over other established methods, including whole-exome sequencing (WES), but still estimated it to only be around 38.6% (Nurchis et al. 2023). This contributes to the increasing pool of evidence that variants in noncoding regions contribute to rare-disease development (Turro et al. 2020; Whiffin et al. 2020). Consequently, even with WGS as a first-line test, the fraction of patients receiving a diagnosis remains low. Possible explanations for this low yield could be insufficient annotation of noncoding variants, errors in the analysis pipeline, including mapping and variant calling, and misassemblies of the reference genome. These issues could be addressed by applying an improved reference genome, but to what extent T2T-CHM13 improves detection of rare and deleterious variants over the established reference genomes GRCh37 and GRCh38 remains to be investigated.

SweGen is a cross-sectional cohort from Sweden providing a resource of genetic variability in the local population (Ameur et al. 2017). It consists of 1000 individuals who have undergone whole-genome sequencing. Since its inception in 2017, SweGen has facilitated clinical genomics research and diagnostics by providing a representative set of genetic variation in healthy individuals and, for example, by serving as a control cohort in SWEDEGENE, a study that has identified genetic variants causing serious adverse drug reactions (Hallberg et al. 2020). In another study, transposable elements (TEs) in protein-coding genes have been characterized in SweGen and used to diagnose two unsolved cases of rare diseases (Bilgrav Saether et al. 2023). SV frequency data from SweGen have been used to map complex chromosomal rearrangements (Eisfeldt et al. 2019). Several studies have used the frequency data from SweGen for identifying potentially pathogenic rare mutations usually not found in healthy individuals, for example, to identify germline mutations associated with breast cancer risk (Helgadottir et al. 2021) and somatic variation in relapsed pediatric acute lymphoblastic leukemia (Sayyab et al. 2021).

In this study, we aim to evaluate the improvement of mapping short-read WGS data to the new T2T-CHM13 reference compared to the reference genomes that are currently used in clinical decision tools. We remapped the WGS data from 1000 individuals from SweGen and compared the mapping, variant calling, and annotation performance, as well as extrapolated what impact a transition from the current reference to the T2T-CHM13 will have for clinical diagnostics. We also provide an open resource with updated summary data for variants in the SweGen cohort based on the T2T-CHM13 reference to be used for research and clinical applications.

## Results

We obtained the original WGS alignments from SweGen, which were BAM files mapped to GRCh37 and contained all generated reads, including those that were not mapped. We reanalyzed the SweGen data set with the freely available nf-core/sarek pipeline using BWA-MEM to T2T-CHM13 v2.0 (Md et al. 2019; Garcia et al. 2020). We performed variant calling using GATK HaplotypeCaller in joint germline calling mode (Van der Auwera and O'Connor 2020). The same data had also been analyzed using GRCh38.p13, so that we were able to leverage the results from that analysis for comparison with our results.

### T2T-CHM13 improves quality of alignments

**Figure 1.**
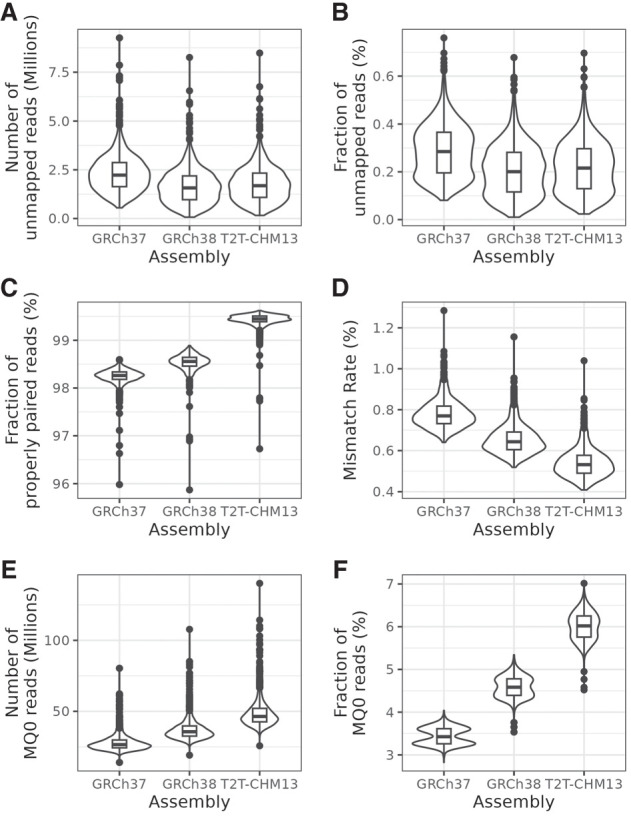
General mapping statistics for all 1000 samples. (*A*) Distribution of the total number of unmapped reads. (*B*) Percentage of unmapped reads per individual. (*C*) Percentage of mapped read pairs that were properly paired, that is, both mates were mapped with the expected distance and orientation. (*D*) Mismatch rate, that is, the fraction of mapped bases that did not match the reference. (*E*) Number of mappings with quality 0, that is, not mapping uniquely. (*F*) Percentage of mappings that had quality 0. MQ0 = mapping quality 0.

In comparison to GRCh37, the number of mapped reads increased by, on average, 558,955 reads per individual, which corresponded to 0.0692% (Fig. 1A,B; Supplemental Table S1). On the other hand, fewer reads were mapped when comparing to GRCh38, with a mean difference of 128,094 per individual, corresponding to 0.0158% of all reads (Fig. 1A,B; Supplemental Table S1). However, we observed that a larger portion of read pairs were properly paired according to BWA-MEM when using T2T-CHM13 compared to the other two references (Fig. 1C; Supplemental Table S1). BWA-MEM considers a read pair properly paired if it consists of two reads where one mate is mapped in forward and the other in reverse direction, both mates’ read directions point toward each other, and the distance between mates does not deviate too much from the mean insert size. In this case, this threshold is approximately six to seven standard deviations from the mean insert size. On average, 99.4% of reads per individual were paired properly (IQR: 99.4%–99.5%) when using T2T-CHM13 (Fig. 1C). In comparison, 97.9% of read pairs had proper orientation when using GRCh37 and 98.2% when using GRCh38. Furthermore, the alignments to T2T-CHM13 showed a lower per-sample mismatch rate (Fig. 1D; Supplemental Table S1). The mean mismatch rate was 0.54% (IQR: 0.48%–0.58%) for T2T-CHM13, compared to 0.78% (IQR: 0.73%–0.82%) for GRCh37 and 0.65% (IQR: 0.60%–0.69%) for GRCh38. This means that reads were mapped to T2T-CHM13 with higher accuracy.

### Alignments previously considered unique are ambiguous

We noticed an increase in the number of alignments that received mapping quality 0 (MQ0)—that is, they mapped equally well to more than one position (Fig. 1E)—as well as a higher percentage of mapped reads with MQ0 (Fig. 1F) with the T2T-CHM13 reference (Supplemental Table S1). MQ0 reads are also significantly overrepresented in SDs (χ^2^ *P*-value < 2.2 × 10^−16^). Overall, 7.98% of all reads were assigned MQ0, but among those that map to SDs, 31.9% were assigned MQ0. This is likely a result of T2T-CHM13 resolving many SDs that appeared as unique regions in GRCh37 and GRCh38 as well as low-complexity regions not included in GRCh37 and GRCh38.

### Coverage of genes is more uniform

**Figure 2.**
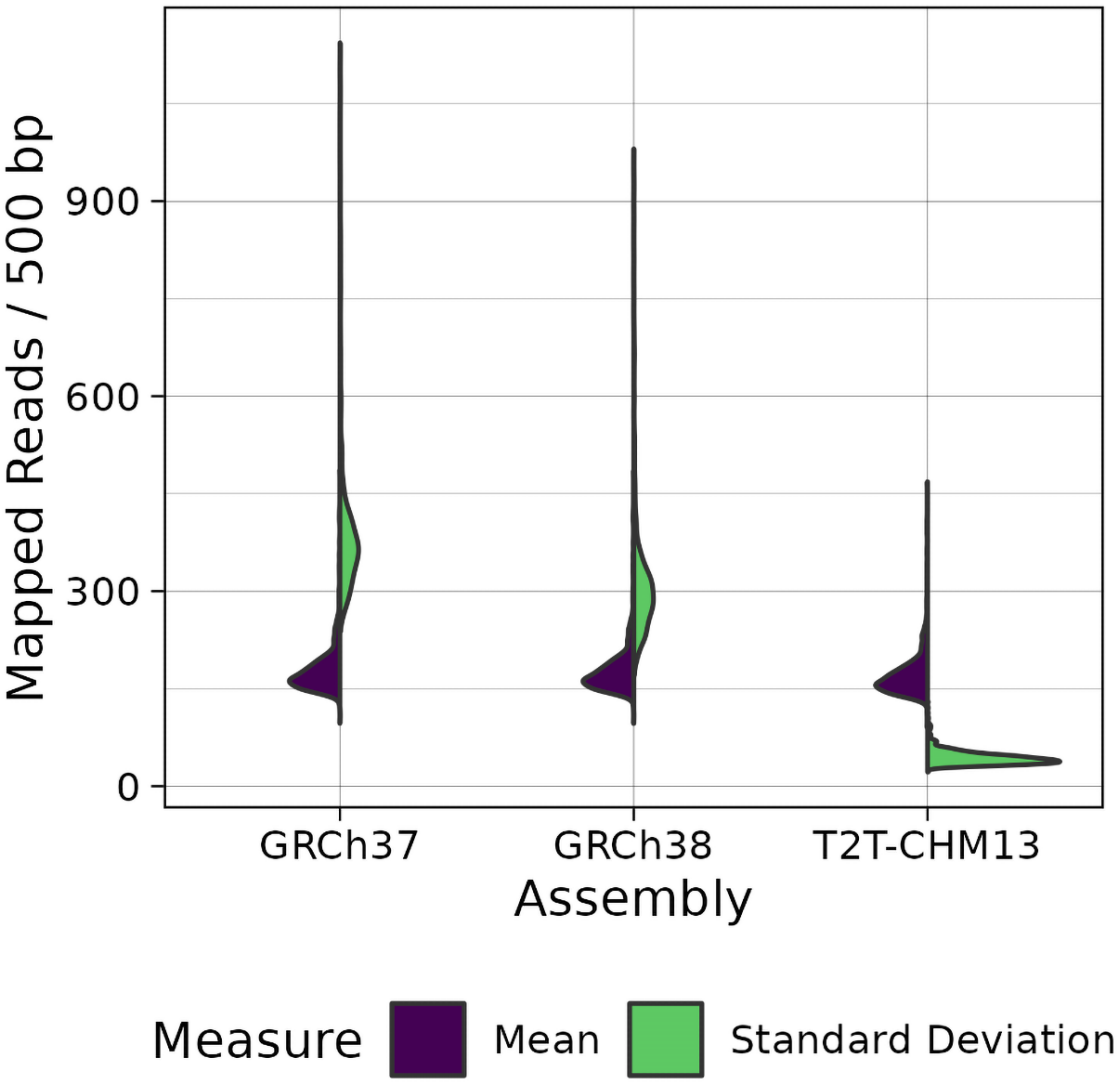
Summary of read depth in gene regions. Genes were divided into nonoverlapping 500-bp bins, and the read depth of each bin was calculated. From these, per-individual means (blue, *left*-facing) and standard deviations (green, *right*-facing) were obtained, which are shown here.

To investigate the coverage of genes in our sequencing data and assess potential improvements to detection of functionally relevant variants, we calculated the read depth for all nonoverlapping 500-bp bins in gene regions annotated by the T2T Consortium. Whereas we did not observe a change in the mean coverage in genes, there was a reduction in the per-individual standard deviation of the read counts compared to GRCh37 and GRCh38, indicating a more uniform coverage of genes when aligning to T2T-CHM13 (Fig. 2).

### Reads from unplaced contigs map mostly to acrocentric short arms

**Figure 3.**
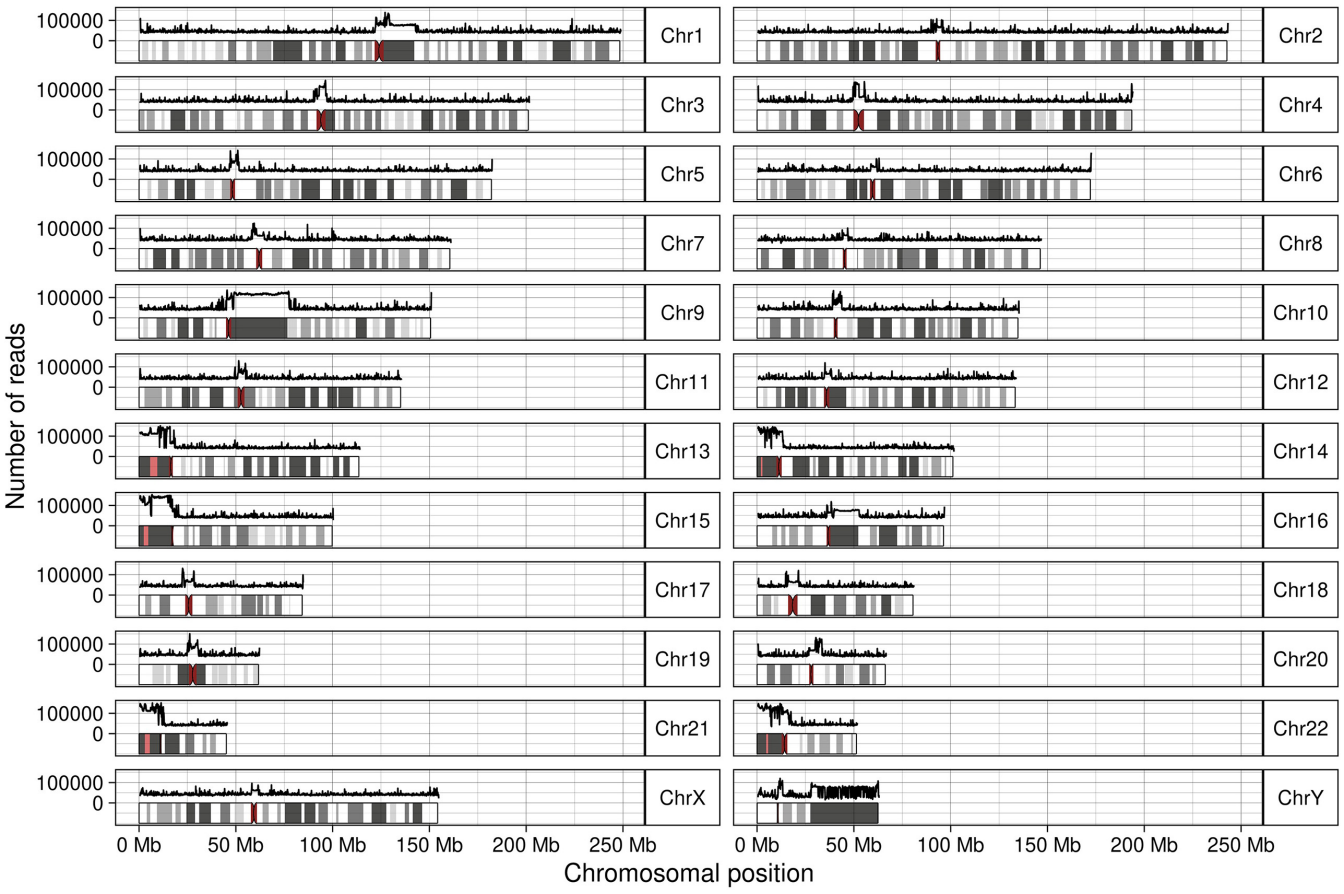
Number of reads from unplaced contigs mapping across T2T-CHM13 in bins of 100 kbp. For each chromosome, an ideogram including the bands is shown; centromeres are dark red and thinner. The *x*-axis corresponds to the position on the chromosome. The *y*-axis is logarithmic and corresponds to the number of reads. The line *above* each chromosome shows the number of reads mapping to a 100-kbp window.

We investigated the mapping positions of reads that were mapped to unplaced contigs in GRCh38. Virtually all reads (99.99974%) that uniquely mapped to unplaced contigs in GRCh38 could be mapped to canonical chromosomes in T2T-CHM13. The short arms of Chromosomes 13, 14, 15, 21, 22, and Y were overrepresented among the regions where these reads mapped, accounting for 84% of reads (Fig. 3; Supplemental Table S2). However, we could only assign a unique canonical chromosome to five unplaced contigs out of 126. Reads from the remaining contigs mapped to positions on multiple chromosomes.

### More rare variants with T2T-CHM13

**Figure 4.**
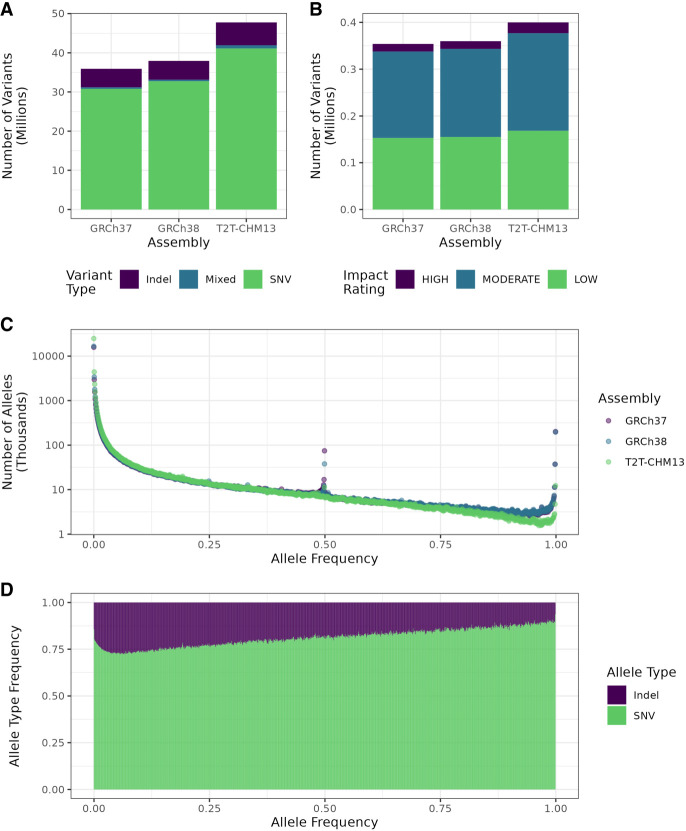
Summaries of variant characteristics from the whole cohort. (*A*) Comparison of overall variant counts, separated by variant type (SNV, indel, or mixed, i.e., both) between variant calls from the different assemblies. (*B*) Overall numbers of variants by predicted impact in the whole call set as determined by SnpEff for each assembly. Variants with impact rating MODIFIER were excluded. (*C*) Distribution of allele frequencies in the three assemblies. (*D*) Fraction of each variant type by allele frequency using T2T-CHM13. Frequencies were rounded to the closest multiple of 0.001 (1/1000).

To assess the general characteristics of the T2T-CHM13 variant calls, we collected statistics about number, frequencies, and types on the cohort level. Overall, we detected 47,744,487 unique high-quality variants in the cohort, of which 19,077,986 (40%) were singletons, an increase from GRCh37 and GRCh38 (Table 1; Fig. 4A). This difference is not explained by the higher number of resolved bases, as the T2T-CHM13 call set shows a higher density of variants overall (15.3 variants/kbp) and singletons in particular (6.12 singletons/kbp) than the older call sets (Table 1). Moreover, we observed higher numbers of rare (allele frequency [AF] < 1%) and low-frequency variants (AF < 5%) (Table 1; Fig. 4C; Supplemental Table S3). On the other hand, the number of common variants (AF ≥ 5%) remained roughly the same, and the number of nonreference alleles with an AF > 50% was generally lower with T2T-CHM13 than with the other references (Fig. 4C). SweGen was established as a resource to capture genetic variability within the Swedish cohort, and it has been shown that these samples cluster closely with other European populations, although they are distinct from central European populations (Ameur et al. 2017). Because T2T-CHM13 matches European ancestries more closely than GRCh37 and GRCh38 (Aganezov et al. 2022), we would expect to observe fewer variants where the nonreference allele is the major allele in our cohort when T2T-CHM13 is used as the reference. Noticeable is also the peak of variants with AF ≈ 0.5 with GRCh37 and GRCh38 (Fig. 4C) which was not seen with T2T-CHM13. When removing variants that deviated strongly from Hardy-Weinberg equilibrium (*P* < 10^−20^), this peak disappeared (Supplemental Fig. S1), which points to it being caused by previously unresolved segmental duplications that give rise to stretches of heterozygous variant calls in GRCh37 and GRCh38.

**Table 1.** General cohort-level characteristics of alignments and variant calls with T2T-CHM13 in comparison with previous assemblies

	T2T-CHM13	GRCh37	GRCh38
Ungapped length (bp)	3,117,275,501	2,861,327,195	2,937,639,396
Avg. % coverage (min/max)	39.0 (20.3/105.9)	39.8 (21.3/107.7)	40.2 (21.5/108.7)
Number of variant sites^a^	47,744,487	35,571,130	37,938,450
Variant rate (variants/kbp)^a^^,^^b^	15.3	12.4	12.9
Number of SNV sites (not in dbSNPv155)	41,931,920 (10,806,250)	30,866,176 (14,806,371)	33,133,695 (12,070,889)
Number of indel sites (not in dbSNPv155)	6,650,809 (3,709,821)	5,089,447 (3,170,927)	5,221,969 (2,457,457)
T_s_/T_v_ ratio	1.50	1.95	1.80
Number of common variants (MAF ≥ 5%)^c^	9,468,024	9,800,121	9,832,496
Number of low-frequency variants (MAF < 5%)^c^	43,151,191	29,714,010	31,895,019
Number of rare variants (MAF < 1%)^c^	37,402,076	24,807,638	26,741,004
Number of singletons (per kbp) [% of total variants]	19,077,986 (6.12) [40.0]	11,502,698 (4.02) [32.3]	13,547,273 (4.61) [35.7]
Number of nonreference alleles with AF > 50%	1,940,331	2,424,122	2,465,936
Number of LoF variants (% of total variants) [in ClinVar genes]	17,446 (0.037) [17,297]	12,628 (0.036) [12,326]	12,829 (0.034) [12,517]
Number of variants with low/medium/high impact (% of total variants)	168,420/208,625/22,848 (0.353/0.437/0.048)	153,001/184,694/16,392 (0.426/0.514/0.045)	155,088/188,347/16,542 (0.409/0.496/0.044)

(T_s_) transition, (T_v_) transversion, (MAF) minor allele frequency, (AF) allele frequency, (LoF) loss of function.

^a^Multiallelic variants were only counted once.

^b^Based on ungapped length.

^c^Each alternative allele was counted separately. If an alternative allele had a frequency >50%, the reference allele was considered the minor allele.

### Proportions of SNVs and indels remain unchanged

To assess whether T2T-CHM13 affected our power to call SNVs or indels, we compared the numbers and proportions of these variant types between references and in previously unresolved regions. Around 87.8% of all called variants were SNVs, which is similar to what was found for GRCh37 (86.7%) and GRCh38 (87.3%) (Fig. 4A). The overall fraction of indels remained roughly the same as well, constituting 13.9% of variants in T2T-CHM13, 14.3% in GRCh37, and 13.7% in GRCh38. In general, low-frequency variants skewed more toward indels and mixed variation (i.e., indels and SNVs at the same site) than common variants in T2T-CHM13 (Fig. 4D). Indels were generally skewed toward deletions in all assemblies. Overall, more bases were deleted than inserted, resulting in an excess of deleted bases of 11,028,492 in T2T-CHM13, which was an increase from GRCh37 (1,065,087) and GRCh38 (816,639) (Supplemental Fig. S2). This excess was also visible in the number of 1-bp deletions, which accounted for 1,425,614 variants. Of these deletions, 729,804 were situated in genes, independent of predicted impact. One-base pair deletions were more likely to be singletons than variants in general; 43.4% (619,334) were private to one individual, as opposed to 40% overall, but less likely to be fixed in the cohort (0.004% [170] vs. 0.036% [16,967] overall).

### Higher numbers of loss-of-function (LoF) variants and variants with predicted functional impact with T2T-CHM13

To confirm our previous assessment of improved detection of variants with functional impact due to better gene coverage, we predicted variant effects using SnpEff, including loss of function. We observed an increased number of variants with an impact rating, that is, a prediction of having impact on the protein function by, for example, causing truncation, loss of function, or triggering nonsense mediated decay (Fig. 4B). Overall, using T2T-CHM13, we identified 474,050 variants with an impact rating of “low,” “moderate,” or “high,” corresponding to ∼0.993% of all called variants. For comparison, 354,087 variants called with GRCh37 (0.996%) and 359,977 called with GRCh38 (0.949%) received an impact rating. Among the variants with an impact rating with T2T-CHM13, 48.4% and 45.2% of variants were predicted to have moderate and low impact, respectively, and the remaining 6.4% were rated high impact (Fig. 4B). Our results thus suggest that improved gene coverage facilitates detection of functionally relevant variation.

**Figure 5.**
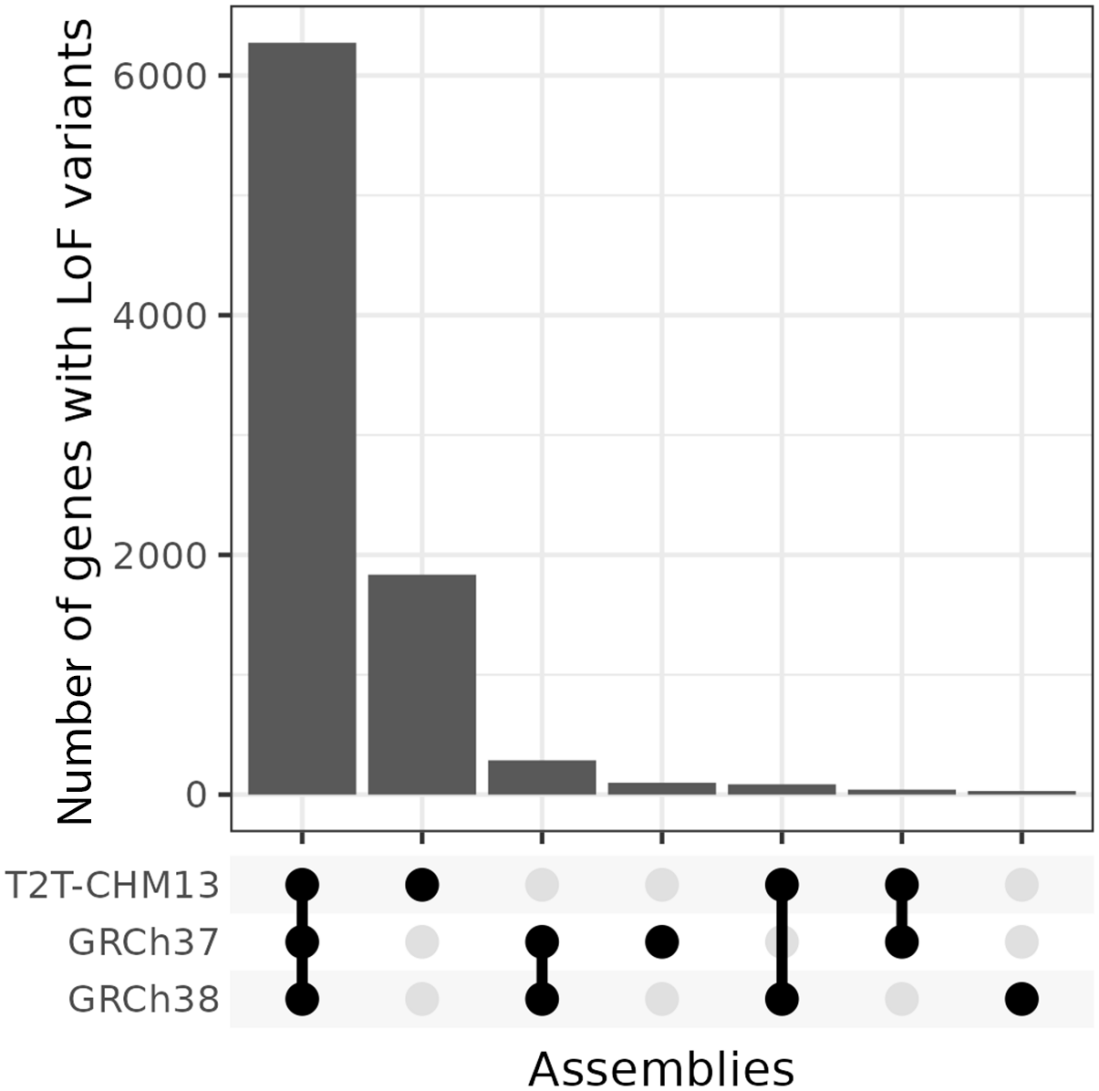
Number of genes with predicted LoF variants shared among assemblies.

We identified 17,446 LoF variants using T2T-CHM13, an increase from GRCh37 and GRCh38 (Table 1). Of these, 17,297 variants were annotated to 8287 genes with existing ClinVar annotations. Most affected genes were shared among all three references (Fig. 5). Across all three references, *MUC4*, which encodes mucin 4, cell surface associated, was the gene with the highest number of LoF variants (Table 2; Supplemental Table S4). However, the majority of LoF variants in *MUC4* fall into a 48-bp variable tandem repeat in exon 2, which is problematic for short reads and might have led to an inflated number of variants. Tandem repeats are a general characteristic of the mucin gene family, which made up five of the 10 top affected genes across all assemblies (Ferez-Vilar and Hill 1999). Additionally, *HLA-DRB1*, belonging to the human leukocyte antigen (HLA) gene family, was present across all references. Another *HLA* gene, *HLA-DRB5*, was among the top 10 for GRCh37 and GRCh38 but not T2T-CHM13. However, as with the mucin gene family, *HLA* genes tend to be difficult to analyze with short reads because they are highly polymorphic.

**Table 2.** Top 10 ClinVar genes by number of loss-of-function variants by assembly

GRCh37		GRCh38		T2T-CHM13	
Gene	Number of LoF variants	Gene	Number of LoF variants	Gene	Number of LoF variants
*MUC4*	143	*MUC4*	172	*MUC4*	245
*MUC19*	99	*MUC3A*	127	*MUC19*	61
*MUC3A*	71	*MUC19*	100	*MUC2*	54
*MUC16*	48	*MUC6*	47	*MAGEC1*	46
*MUC6*	43	*HLA-DRB1*	39	*MUC5AC*	46
*HLA-DRB1*	37	*MUC16*	36	*HLA-DRB1*	35
*HLA-DRB5*	29	*HLA-DRB5*	33	*AHNAK2*	30
*ZNF717*	23	*ZNF717*	25	*MUC17*	29
*PDE4DIP*	22	*ZNF880*	19	*NACA*	28
*ANKRD36*	21	*GOLGA6L6*	18	*IGFN1*	26

(LoF) loss of function.

With these considerations in mind, we curated a random list of 10 novel LoF variants which were called in genes which had no such variants in GRCh37 and GRCh38. We could confirm the existence of six but not the other four by visual inspection (Supplemental Fig. S3). Previous studies have suggested that an increased number of high-impact predictions are likely to represent artifacts arising from lower quality or incomplete assemblies (Cooper and Shendure 2011). It is, therefore, plausible that some of the novel LoF variants we did not curate are artifacts rather than true LoF variants, particularly given the improved quality and completeness of the T2T-CHM13 assembly. Moreover, we validated these LoF variants in two individuals (SweGen0945 and SweGen0970) with available de novo assemblies from 75× Pacific Biosciences (PacBio) continuous long-read (CLR) sequencing, where we could confirm 15 of 130 (11.5%) and 16 of 139 (11.5%) variants, respectively (Supplemental Table S5A,B). For a third individual (SweGen0969), we could make use of available 35× WGS PacBio CLR data and confirm 21 of 143 variants (14.7%) (Supplemental Table S5C).

Among the top 10 affected genes in T2T-CHM13, only four did not belong to the aforementioned gene families. MAGE family member C1 (*MAGEC1*) has close links to melanoma and myeloma development (Jungbluth et al. 2005; Caballero et al. 2010). AHNAK nucleoprotein 2 (AHNAK2) may play a role in calcium signaling and is upregulated in lung cancer (Komuro et al. 2004; Liu et al. 2020). Nascent-polypeptide-associated complex (NACA) binds to newly created polypeptides to prevent wrong translocation to the endoplasmatic reticulum and plays a role in bone formation and red blood cell differentiation and has been linked to dermatitis (Lauring et al. 1995; Natter et al. 1998; Lopez et al. 2005). Immunoglobulin like and fibronectin type III domain containing 1 (IGFN1) may play a role in skeletal muscle development and has been associated with polypoidal choroidal vasculopathy (Wen et al. 2018; Cracknell et al. 2020).

### Previously unresolved regions are not enriched for singletons

To assess whether the observed increase in singleton calls was driven by regions that were unresolved in GRCh37 and GRCh38, we collected general variant call statistics restricted to these regions. There were 8,321,170 variants in regions unresolved in GRCh37, and in regions which were unresolved in GRCh38, we identified 7,826,115 variants (Table 3). Within these regions, we observed a higher density of variants (unresolved in GRCh37: 30.9 variants/kbp; unresolved in GRCh38: 31.1 variants/kbp) than in the remaining genome (13.9 variants/kbp). From that, we can conclude that these variants contribute to a majority, but not all, of the excess in the number of variants called with T2T-CHM13. This was also apparent in unique regions syntenic between GRCh38 and T2T-CHM13, where T2T-CHM13 showed a higher variant density (13.9 variants/kbp) than GRCh38 (12.5 variants/kbp).

**Table 3.** Overall characteristics of variant calls in previously unresolved regions

	Regions unresolved in		All of
	GRCh37	GRCh38	T2T-CHM13
Total length (bp)	268,965,814	251,330,203	3,117,275,501
Number of variant sites^a^	8,321,170	7,826,115	47,744,487
Variant rate (variants/kbp)^a^	30.9	31.1	15.3
Number of SNV sites	7,710,249	7,265,225	41,931,920
Number of indel sites	829,457	771,429	6,650,809
Number of common variants (MAF ≥ 5%)^b^	695,892	621,512	9,293,456
Number of low-frequency variants (MAF < 5%)^b^	8,670,575	8,211,562	43,103,217
Number of rare variants (MAF < 1%)^b^	7,721,885	7,316,843	37,800,790
Number of singletons (% of total variants)	2,540,127 (30.5)	2,335,181 (29.8)	19,077,986 (40.0)
Number of LoF variants	185	99	17,446
Number of variants with low/medium/high impact (% of total variants)	1,435/2,102/286 (0.017/0.025/0.003)	719/1,048/155 (0.009/0.013/0.002)	168,420/208,625/22,848 (0.353/0.437/0.048)

(MAF) minor allele frequency, (LoF) loss of function.

^a^Multiallelic variants were only counted once.

^b^Each alternative allele was counted separately. If an alternative allele had a frequency >50%, the reference allele was considered the minor allele.

**Figure 6.**
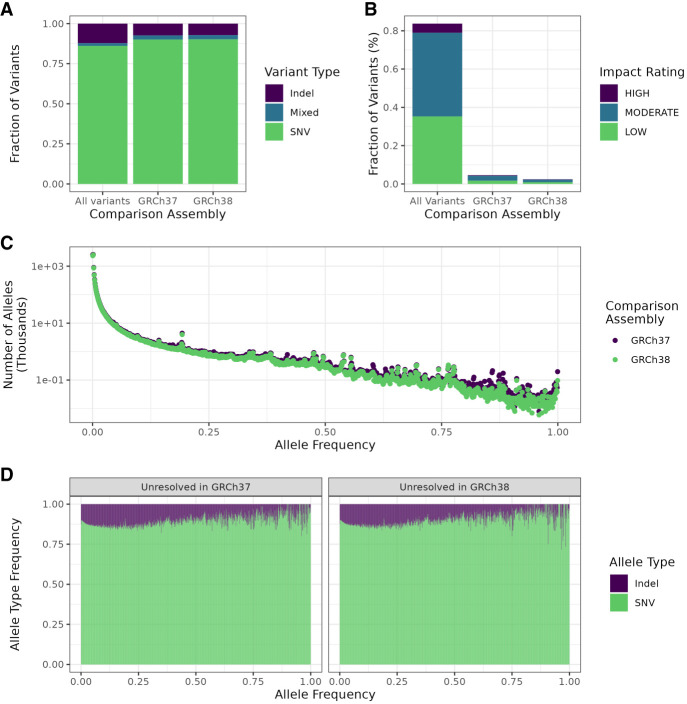
Summaries of variant characteristics from the whole cohort, restricted to newly resolved regions. (*A*) Fraction of variants by type in the novel regions in the T2T-CHM13 assembly that were unresolved in the reference as shown on the *x*-axis and the whole genome. (*B*) Fractions of variants by predicted impact in the whole call set as determined by SnpEff in previously unresolved regions and the whole genome. Variants with impact rating MODIFIER were excluded. (*C*) Distribution of allele frequencies for T2T-CHM13 specific variants mapped to regions not resolved in the two previous assemblies. (*D*) Fraction of each variant type by allele frequency. Frequencies were rounded to the closest multiple of 0.001 (1/1000).

Variants in regions that were previously unresolved in GRCh37 and GRCh38 were more biased toward SNVs (which made up 92.6% and 92.8% of variants there, respectively) than other regions (Fig. 6A). However, the density of deletions was higher in these regions (unresolved in GRCh37: 2.45 deletions/kbp; unresolved in GRCh38: 2.46 deletions/kbp) than in the rest of the genome (1.65 deletions/kbp). We detected an excess of 4,705,752 deleted bases in regions which were unresolved in GRCh38, (4,947,747 bp in regions not in GRCh37) despite only contributing 13% of all indels. The excess in the regions syntenic with GRCh38 is still higher, however, with 6,322,740 more bases deleted than inserted.

The fraction of singletons was lower than in the genome in general (χ^2^ *P* value < 2.2 × 10^−16^) (Table 3), suggesting that the newly resolved regions do not contribute disproportionately to the number of singletons despite a higher density of singletons (Table 3). However, they were significantly enriched for rare variants (χ^2^ *P* value < 2.2 × 10^−16^) and contributed the majority of the excess of rare alleles that were not observed in GRCh37 (61.1% of 12,594,438) or in GRCh38 (68.6% of 10,661,072).

### Previously unresolved regions contain a low rate of variants with predicted impact on a protein

Focusing on previously unresolved regions, we found that they had a much lower fraction of variants with predicted impact on a protein according to SnpEff. The fractions of variants with at least low impact were significantly lower than those of all variants called with T2T-CHM13. In the regions unresolved in GRCh37, there were 3823 variants with a rating of at least low impact, corresponding to 0.045% (χ^2^ *P*-value < 2.2 × 10^−16^). In the parts of the genome which were unresolved in GRCh38, there were 1922 variants with assigned impact rating, which corresponded to 0.025% (χ^2^ *P*-value < 2.2 × 10^−16^). The distribution of impact ratings among these variants also differed slightly between regions that were unresolved in GRCh37 compared to GRCh38, with a larger fraction of high-impact variants in regions that were unresolved in GRCh37 (Fig. 6B). Together, this suggests that variations in the previously unresolved regions generally have lower impact and might therefore be under purifying selective pressure. However, this difference could also be due to a lower density of genes or a lack of annotations compared to regions that have been available to functional annotation before.

### Proportion of SNVs of per-individual variants decreases with use of T2T-CHM13

**Figure 7.**
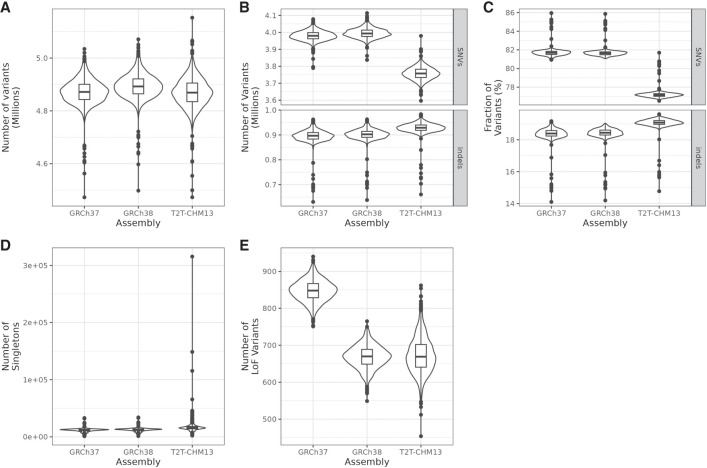
Summaries of several per-individual variant call statistics. Statistics included only variants passing filters. (*A*) Number of overall variants. (*B*) Number of SNVs and indels. (*C*) Fraction of SNVs and indels of called variants. (*D*) Number of singletons. (*E*) Number of LoF variants.

Considering the differences we observed on the cohort level between GRCh37, GRCh38, and T2T-CHM13, we also investigated how variant calls differed at the individual level. Despite the higher total number of called variants in the cohort with T2T-CHM13, we did not observe this increase in the number of variants called per individual (Fig. 7A; Table 4). After limiting the comparison to syntenic regions with high uniqueness in GRCh38 and T2T-CHM13, we observed a slight decrease in the number of variants per individual (mean difference = −212,696.8, *P* value < 2.2 × 10^−16^) and their density (mean difference = −0.039 variants/kbp, *P* value < 2.2 × 10^−16^) (Supplemental Table S6).

**Table 4.** Per-individual statistics of variant calls

	T2T-CHM13	GRCh37	GRCh38
Mean number of variants per individual	4,868,620	4,939,144	4,892,944
Mean number of SNVs (fraction of total variants)	3,757,819 (80.2%)	4,041,524 (81.8%)	3,995,225 (81.6%)
Mean number of indels (fraction of total variants)	928,186 (19.8%)	901,550 (18.2%)	901,818 (18.4%)
Mean number of homozygous variants (IQR)	1,218,295 (1,197,992–1,235,602)	1,491,412 (1,476,977–1,503,380)	1,491,407 (1,476,992–1,503,377)
Mean number of heterozygous variants (IQR)	2,479,761 (2,444,590–2,512,655)	2,490,186 (2,462,633–2,520,624)	2,490,402 (2,462,660–2,520,740)
Mean number of singletons (IQR)	17,034 (14,007–18,263)	11,501 (10,550–13,122)	12,145 (11,181–13,756)
Mean number of LoF variants (IQR)	669 (641–702)	848 (829–867)	668 (649–689)

(IQR) Inter-quartile range, (LoF) loss of function.

We also assessed whether the proportions of SNVs and indels changed when calling variants with T2T-CHM13. The number of SNVs per individual decreased and the number of indels increased slightly (Fig. 7B; Table 4). These changes also became apparent in the fraction of SNVs and indels in all called variants per individual (Fig. 7C). The mean fraction of SNVs decreased, whereas we observed an increased proportion of indels. This discrepancy can be explained by the previously described observation that the fraction of indels tends to be higher among rare variants and that we detected fewer high frequency but a larger number of rare variants. The called indels also tended to skew more toward deletions than the rest of the genome.

### Increased power to detect rare and loss-of-function variants per individual

The number of singletons called per individual was higher with T2T-CHM13 (Table 4; Fig. 7D). There were four individuals with singleton counts of more than three standard deviations above the mean, which we considered outliers. These four individuals were different from the four individuals with the largest number of singletons with GRCh38. However, they made up four of the six individuals with the lowest fraction of properly paired reads and showed anomalies in certain other QC metrics, such as the number of read pairs on different chromosomes. Considering that this was consistent across references, we believe these high numbers of singletons to be caused by technical issues with these four samples, which had not been identified before (Supplemental Table S7). Whereas these individuals accounted for approximately 645,000 singletons, they did not drive the observed increase in per-individual singletons, as every individual in the cohort had an increased number of singleton variants (Supplemental Fig. S4). Furthermore, we observed a slightly increased number of variants with predicted LoF effects per individual over GRCh38 (mean difference = 4.778, *P* value = 0.000722), which agreed with the higher number in the cohort as a whole and showed the benefit of improved sequencing coverage in gene regions (Fig. 7E; Table 4). However, in comparison to GRCh37, the per-individual number of LoF variants actually decreased (mean difference = −174.429, *P* value < 2.2 × 10^−16^). This excess can be explained by variants whose reference alleles were the LoF alleles, which were corrected in GRCh38 and appeared as common or fixed variants in SweGen. For instance, there were 194 LoF variants in the GRCh37 call set with AF > 90%, compared to 83 in GRCh38 and 24 in T2T-CHM13.

## Discussion

In this study, we have shown that using the new T2T-CHM13 reference can improve alignments, variant calls, and sensitivity for rare, singleton, and LoF variants over GRCh37 and GRCh38, which are currently in widespread use, including in genes with clinical relevance. This is an important prospect for clinical genetics as rare, ultra-rare, and de novo variants are often the cause of rare disease. Continuing efforts are aiming to bring sequencing, and especially WGS, into the diagnostic routine because of its power to detect these variants (Tesi et al. 2023). For instance, the UK 100,000 Genomes Project found that genome sequencing significantly increases the diagnostic yield of rare diseases (Smedley et al. 2021). The Genomics England project is finding an increasing number of rare variants with clinical significance and enabling diagnoses of previously undiagnosed patients (Han et al. 2022; Joyce et al. 2022; Vadgama et al. 2022; Kojic et al. 2023). By employing T2T-CHM13 as a reference for first-line WGS in the clinic, the number of people receiving a diagnosis for a rare disease is likely to improve further. Moreover, it is becoming increasingly clear that reanalysis of sequencing data can lead to better diagnoses and improved patient outcomes (Machini et al. 2019; Kingsmore et al. 2022). Through reanalysis, it is possible to detect new causal variations using improved tools and annotation and provide treatment to affected individuals. Considering this, FixItFelix, which realigns reads falling into medically relevant genes using a modified GRCh38 reference with certain genes replaced by their T2T-CHM13 assemblies, was developed (Behera et al. 2023). This tool only requires reanalysis of smaller regions and, as such, provides a faster turnaround. However, it requires limiting the scope of the analysis to manually curated regions and, therefore, is not suitable for a whole-genome study. As such, a re-evaluation of inconclusive clinical samples using T2T-CHM13, including reads that failed to map to canonical chromosomes, can provide valuable diagnoses to patients.

Rare variants are also becoming more prominent in genetic epidemiology. GWASs have only been able to explain part of the observed heritability. Recent studies showed that part of this missing heritability can be explained by rare variants and successfully quantified their effect on complex traits and disease risk (The UK10K Consortium 2015; Höglund et al. 2019; Wang et al. 2021; Kierczak et al. 2022). Consequently, the possibility of detecting more rare variants, especially in regions which lacked an assembly but are functionally relevant, such as centromeres, promises to close the gap in quantifying the missing heritability.

The resources provided by the T2T Consortium, such as lifted-over versions of dbSNP, allow for the adoption of existing workflows to the new reference as well as alignments and variant calls generated using it. However, there is currently a lack of original annotation and databases based on T2T-CHM13. Reliance on annotations generated using older references and lifted over to T2T-CHM13 might negatively impact results, as not all variants can be lifted over. We expect this issue to diminish as more resources, such as this one, become available. Another limitation was that we were unable to unambiguously elucidate why four individuals were outliers with regard to number of singletons. As these four individuals showed some anomalies regarding some QC measures, a possible explanation might have been a higher sensitivity to sample quality or technical issues when aligning to T2T-CHM13. However, we could not conclusively confirm this hypothesis at this point. Furthermore, as the version of T2T-CHM13 we used included unmasked pseudoautosomal regions of Chromosome Y, variants called in these regions suffer from lower accuracy. However, there were only 415 variants with a predicted functional impact, so these issues did not significantly impact our results (Supplemental Table S8). In addition, we did not restrict our analyses to short-read accessible regions. Even though variants outside these regions have a higher risk of being false positives, general trends regarding variant impacts and types within them agree with our findings from the whole genome (Supplemental Table S9; Supplemental Fig. S5).

We observed, in general, improvements in mapping and variant calling performance similar to those presented by the T2T Consortium in their investigation (Aganezov et al. 2022). These included an increase in quality of alignments as both the fraction of properly paired and oriented read pairs increased as well as the mismatch rate decreased. The newly assembled regions also allowed us to identify reads that appeared to map uniquely in older references but that actually map to duplicated regions which were previously unknown. This allowed for greater confidence in the variant calls because spurious variants, which were called as common alleles but originated from separate copies of the same SD, could be removed. Furthermore, common variants might have disappeared because of altered alleles in T2T-CHM13.

On the other hand, we identified generally more variants overall, in terms of absolute number and density, including more deletions, than with previous references, which might be a sign of reference bias associated with the increased number of duplicated sequences in T2T-CHM13. This difference was also present in syntenic regions between GRCh38 and T2T-CHM13 with high uniqueness, where we expected calls to be largely consistent between references. However, at the individual level, we observed a slight decrease in variant number and density in these regions. Therefore, we argue that the majority of variants are called consistently and that the observed opposite trends of these metrics between cohort and individual level are due to minor differences in the syntenic regions and the changes to variant composition mentioned above.

The markedly elevated number of LoF variants in specific genes, many of which are implicated in disease-related pathways, suggests that variation in these genes have been systematically underrepresented in previous reference assemblies. This finding underscores the importance of T2T-based mapping, particularly for diseases where accurate variant detection in these pathways is critical for understanding pathogenesis and improving diagnostic resolution. However, some additional LoF variants we identified, including in syntenic regions, were technical artifacts. However, through our curation of LoF variants in T2T-CHM13 in genes without any detected LoF variation in GRCh37 and GRCh38, and additional validation using PacBio CLR data, which, despite its higher error rate, allowed us to confirm up to 14.7% of novel LoF variants, we could show that there was, in fact, a higher sensitivity for functionally relevant variation. As such, the balance between detection of novel LoF variants and technical artifacts is an important consideration for future projects using T2T-CHM13, especially considering that, as before, LoF variants should be confirmed experimentally before they can be used to inform clinical decisions.

In conclusion, the T2T-CHM13 reference provides a valuable resource that gives improved alignments and variant calling. Based on our results, we recommend, going forward, the adoption of T2T-CHM13 for novel sequencing studies. Re-evaluation of sequenced samples, especially inconclusive ones, using T2T-CHM13 can provide further insights into genetic variation, including high-impact events that might otherwise be missed. Considering the computational complexity of remapping and recalling large cohorts, it can be sensible to take an iterative approach. However, as more resources based on T2T-CHM13 become available, we can expect increased power and precision in statistical and clinical genetics applications.

## Methods

### The SweGen cohort

The SweGen cohort is a cross-sectional cohort of the Swedish population consisting of 1000 individuals that all passed quality control as described in the original SweGen paper (Ameur et al. 2017). The majority of SweGen comprises 942 unrelated individuals from the Swedish Twin Register (STR) who were selected to mirror the overall distribution of genetic principal components (PCs) of all 10,000 STR individuals that underwent SNV genotyping (Lichtenstein et al. 2002). The remaining 58 individuals were selected from the Northern Swedish Population Health Study (NSPHS) (Igl et al. 2010). NSPHS individuals were selected based on their genetic PCs as well. Library preparation and WGS were responsibility of Sweden's National Genomics Infrastructure (NGI) in Stockholm (NGI-S) and Uppsala (NGI-U). After fragmenting to 350-bp inserts and library preparation, DNA samples underwent WGS on the Illumina HiSeq X platform using v2.5 chemistry (Ameur et al. 2017). The 1000 individuals included in the data set all passed QC. The original mappings were obtained by aligning the raw reads to GRCh37.p13 using BWA-MEM 0.7.12. The mappings underwent quality control, which was described previously, including base quality recalibration and duplication marking (Ameur et al. 2017). In addition, alignments and variant calls based on GRCh38.p14 including alternative haplotypes and decoys were available. The final GRCh37 BAM files served as the input for our analysis pipeline as they contained all reads, including unmapped ones, and allowed us to take advantage of the already performed quality control.

### Remapping and variant calling

We obtained the T2T-CHM13 reference genome version 2.0, a lifted-over version of dbSNP and a BED file of segmental duplications from the T2T Consortium's GitHub page (https://github.com/marbl/CHM13). This version of the reference genome contains an assembly of Chromosome Y and softmasked repeats. We performed mapping and variant calling using nf-core/sarek version 3.2.0, which uses GATK 4.3.0.0, with default parameters corresponding to GATK best practices (McKenna et al. 2010; Di Tommaso et al. 2017; Ewels et al. 2020; Garcia et al. 2020). Shortly thereafter, the reads contained in the delivered BAM files, that is, the post-QC alignments to GRCh37p13, which contained all generated reads, even those that failed original QC or were not aligned, underwent read QC using FastQC 0.11.9 (https://github.com/s-andrews/FastQC?) and fastp 0.23.2 (Chen et al. 2018). Reads failed QC if more than 40% of bases had a quality score below 15, or more than five base calls were indeterminate, that is, they were called as “N.” On average, 4.1% of reads (inter-quartile range [IQR]: 3.6%–4.5%) failed this filter. The remaining reads were aligned using BWA-MEM version 0.7.17 (Md et al. 2019). The resulting CRAM files were merged, sorted, and indexed using SAMtools 1.16.1 (Li et al. 2009).

Variants were called using GATK HaplotypeCaller with the joint germline calling feature enabled. For Variant QC, GATK VariantRecalibrator was used with lifted-over versions of dbSNPv155, 1000 Genomes Omni 2.5 variants, as well as 1000 Genomes phase I and gold standard indels. We included all variants passing the more lenient default threshold (truth sensitivity 99.9%–100%) for increased sensitivity and because those were available in the call sets of the older assemblies. General statistics on the performance of the T2T-CHM13 reference were based on the reports automatically generated by SAMtools and BCFtools 1.17 as part of the sarek pipeline (Li 2011; Danecek et al. 2021). We added annotations using BCFtools and as well as functional effect predictions, such as loss of function using SnpEff based on the lifted-over version of dbSNP and curated transcripts from RefSeq 5.1 provided by the T2T Consortium (Cingolani et al. 2012a,b). We created a subset of putatively clinically relevant LoF variants by filtering for annotation to genes in ClinVar (version 20240215) (Landrum et al. 2014). We identified variants in previously unresolved regions, both in relation to GRCh37 and GRCh38, by subsetting the joint VCF with BCFtools using the “CHM13 unique” track in the UCSC Genome Browser (Kent et al. 2002). We obtained already existing alignments to GRCh37 and GRCh38 from the SweGen data set as well as variant calls, both per-individual and joint calls for the whole cohort, generated from these alignments. We generated mapping and variant reports using SAMtools and BCFtools. We calculated read depth in gene regions by creating nonoverlapping 500-bp bins covering these regions. Additionally, we created a subset of variants expected to be consistent between GRCh38 and T2T-CHM13 by subsetting for syntenic regions and the “UMAP S100” track from the UCSC Genome Browser, as these regions are consistent between assemblies and allow for high-confidence short-read mapping (Karimzadeh et al. 2018).

### Validation of putative LoF variants

We visually inspected 10 randomly selected predicted LoF variants from genes that had no reported LoF variants in the GRCh37 and GRCh38 call sets in Integrative Genomics Viewer (IGV) 2.8.13 (Robinson et al. 2011). We validated putative LoF variants in the same set of genes in individuals SweGen0945 and SweGen0970 using available de novo assemblies generated from 75× coverage PacBio continuous long read sequencing (Ameur et al. 2018). We mapped the assembled contigs to T2T-CHM13 using minimap2 2.26 and called variants using the included version of paftools (Li 2018). We compared the variants found in the de novo assemblies with those in the short reads using RTG Tools vcfeval 3.12.1 (https://github.com/RealTimeGenomics/rtg-tools), restricting the analysis to regions deemed reliable for variant calling by paftools, that is, covered by exactly one contig, excluding alignments shorter than 10 kbp (Cleary et al. 2015).

We validated putative LoF variants in individual SweGen0969 using previously generated PacBio CLR sequencing data (Schmitz et al. 2023). We aligned the reads to T2T-CHM13 using minimap2 version 2.26 and called variants using Longshot 1.0 (Edge and Bansal 2019). We compared the variants found in the PacBio data with those in the short reads using RTG Tools vcfeval 3.12.1, restricting the analysis to regions with coverage higher than 15× and lower than 60× to exclude copy-number variable regions.

### Ethics declaration

All participants in the Swedish Twin Register gave informed consent, and the study was approved by the regional ethics committee in Stockholm (Regionala Etikprövningsnämnden, Stockholm, dnr 2007-644-31 and 2014/521-32). Participants of the Northern Swedish Population Health Study gave their informed consent, and the study was approved by the regional ethics committee in Uppsala (Regionala Etikprövningsnämnden, Uppsala, dnr 2005:325 and 2016-03-09).

## Supplemental Material

Supplement 1

Supplement 2

## Data access

All data generated in this study are available at FEGA Sweden and have been submitted to the European Genome-phenome Archive (EGA; https://ega-archive.org) under accession number EGAS50000000906. Cohort-level allele frequencies and variant annotation are publicly available on the SweGen website (https://swefreq.nbis.se/dataset/SweGen) and at Zenodo (https://doi.org/10.5281/zenodo.17209718).
