# Supplementary material for: T2T-CHM13 improves read mapping and detection of clinically relevant genetic variation in the Swedish population
Source: Genome Res. 2025 Nov;35(11):2377–88. doi: 10.1101/gr.279320.124 (PMC12581843; doi:10.1101/gr.279320.124)

Supplemental Figures

# Supplemental Figure S1

Distribution of allele frequencies (AFs) of all variants and variants not violating Hardy-Weinberg-Equilibrium (HWE) ($P>{10}^{20}$) called using GRCh38. The peak around AF=0.5 disappears when removing variants violating HWE, showing that most of the excess of variants were artifacts


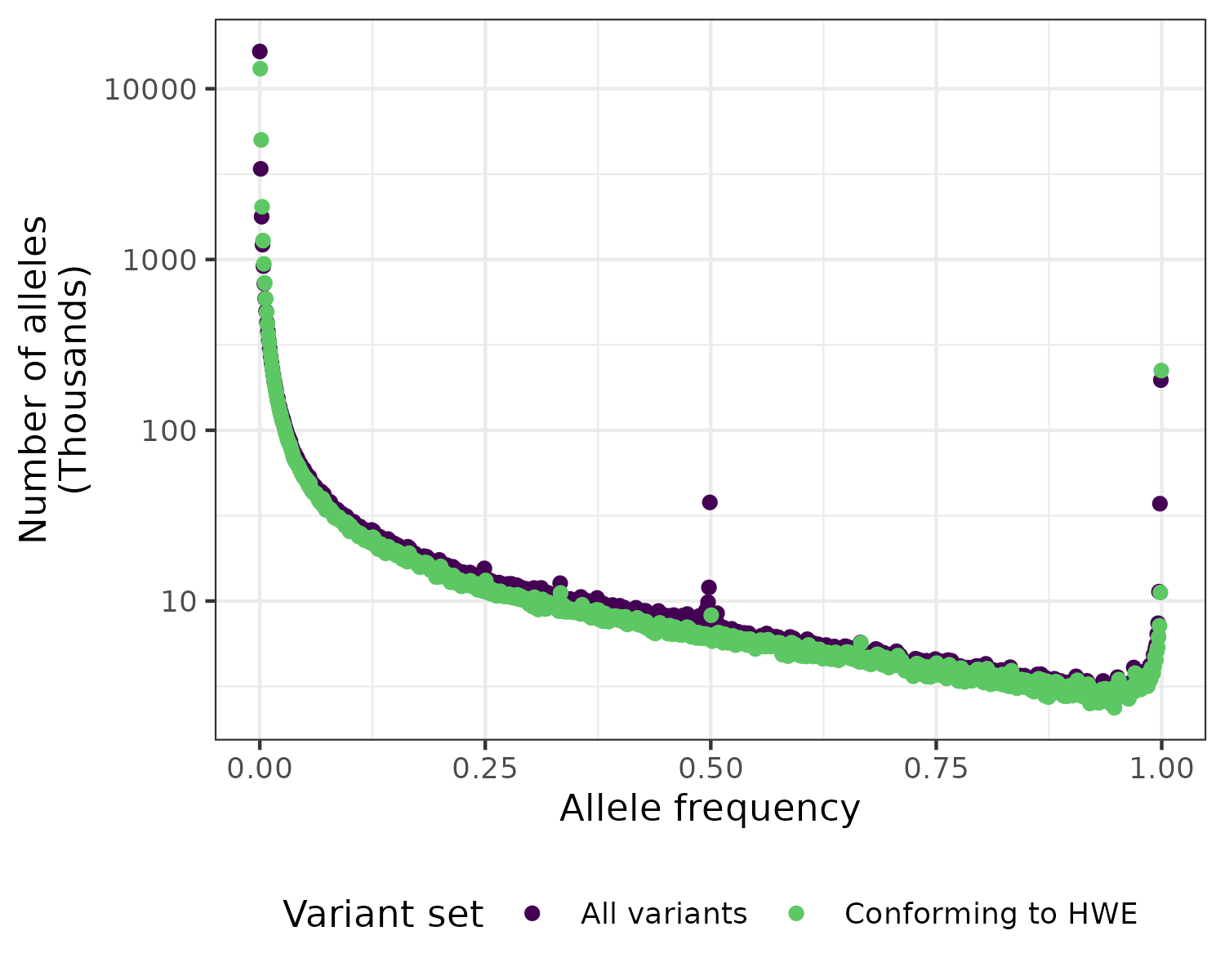


# Supplemental Figure S2

Distribution of indels in all three assemblies by length. Deletions have negative length. Lengths 60, -60 include variants longer than 60 bp.
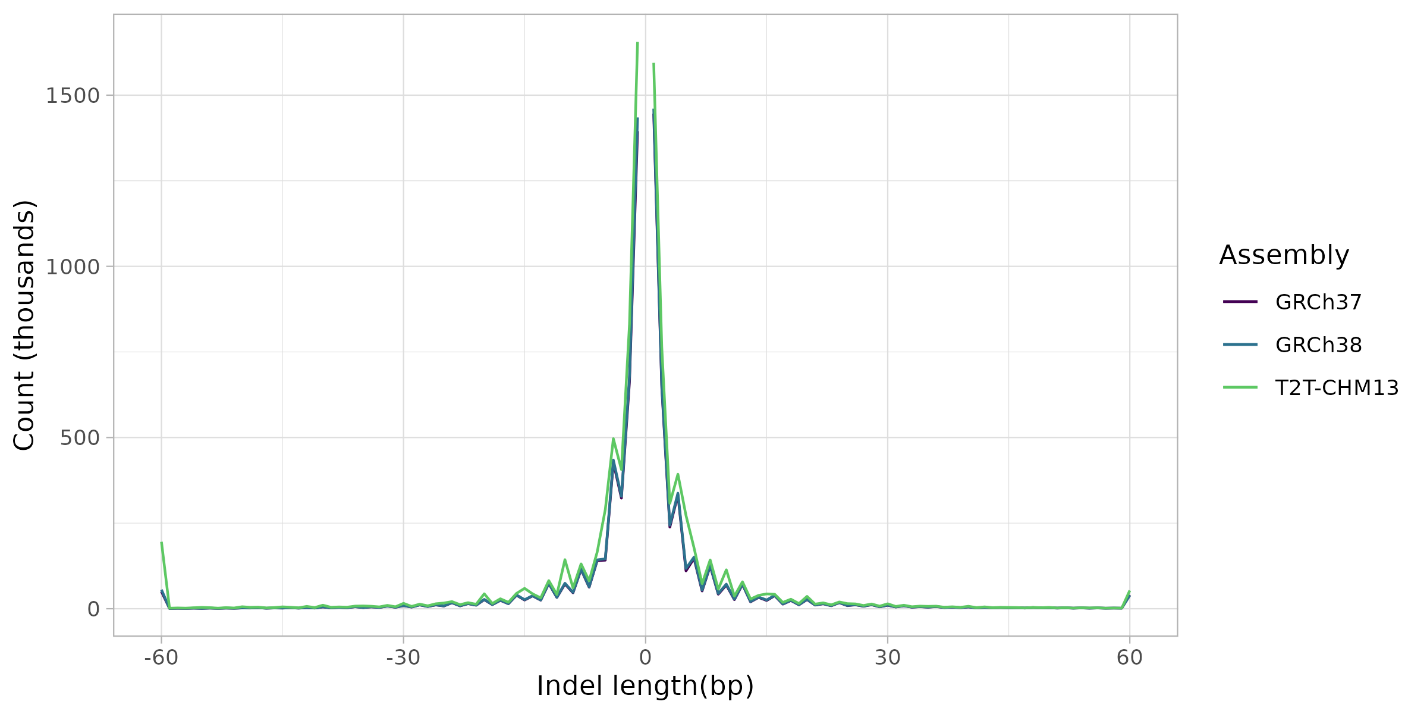


# Supplemental Figure S3

Curation of ten random novel predicted LoF variants.

Reads are sorted and colored by strand. “Realigned” refers to HaplotypeCaller’s BAM output.

## Supplemental Figure S3a: Variant 1: chr1:21491738C>T

Is also a singleton variant. Shows up well in both strands. Depth is normal at 42. Can be considered true.


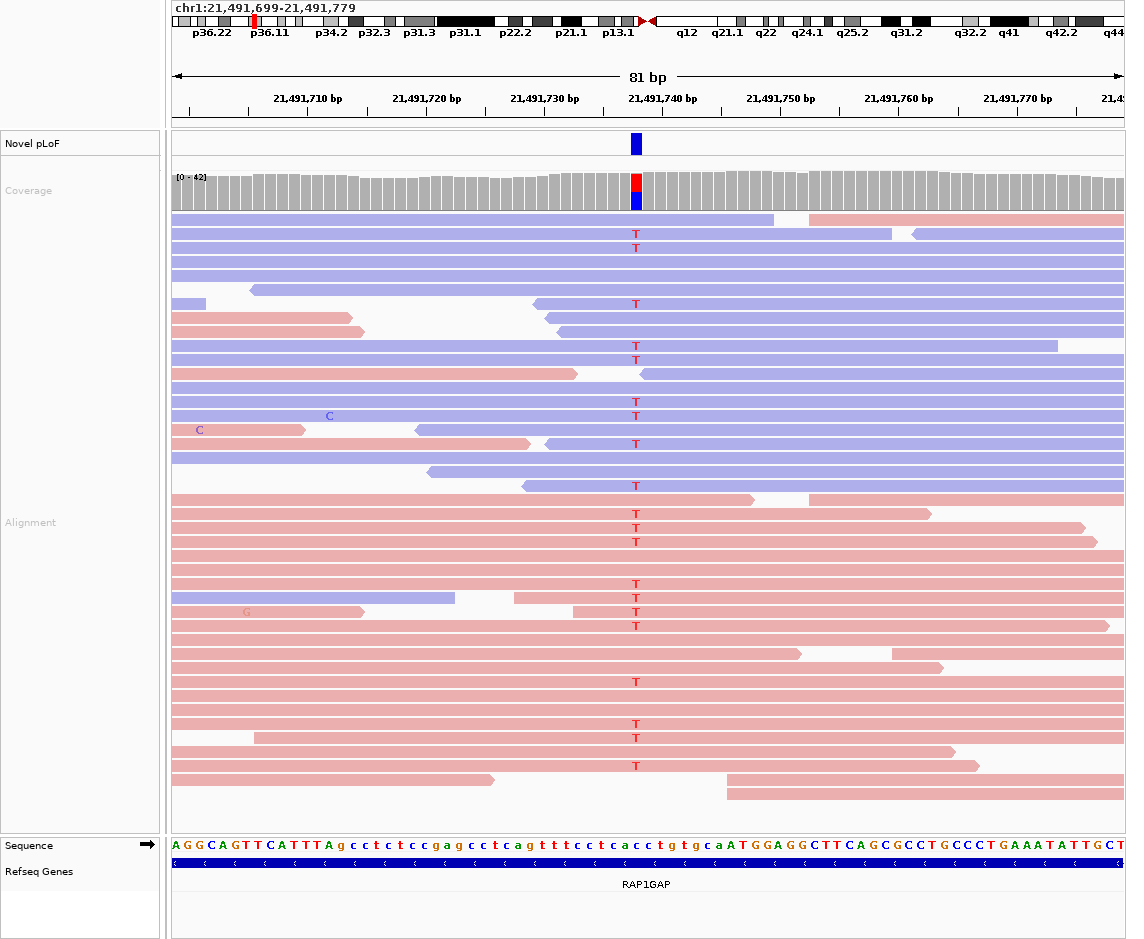


## Supplemental Figure S3b: Variant 2: chr1:151233622TCC>*,T


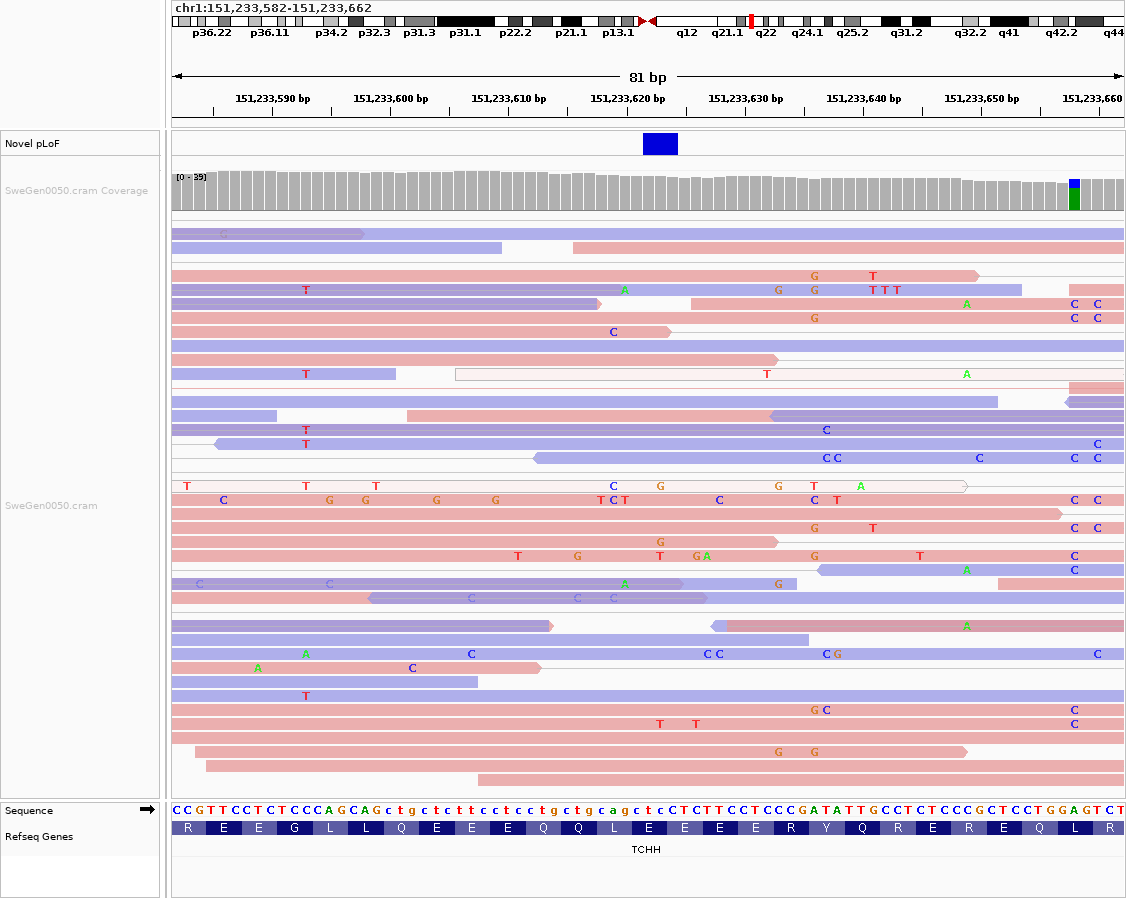
Called in 28 individuals. Not immediately visible in alignment (top). However, HaplotyeCaller’s realigned BAM shows deletion in 69 (middle). Coverage is slightly lower (25). Also biased towards one strand. In 146, larger deletion on either strand (bottom). Coverage at 24 around deletion. T allele might be artifact. The * allele is probably part of a larger deletion.


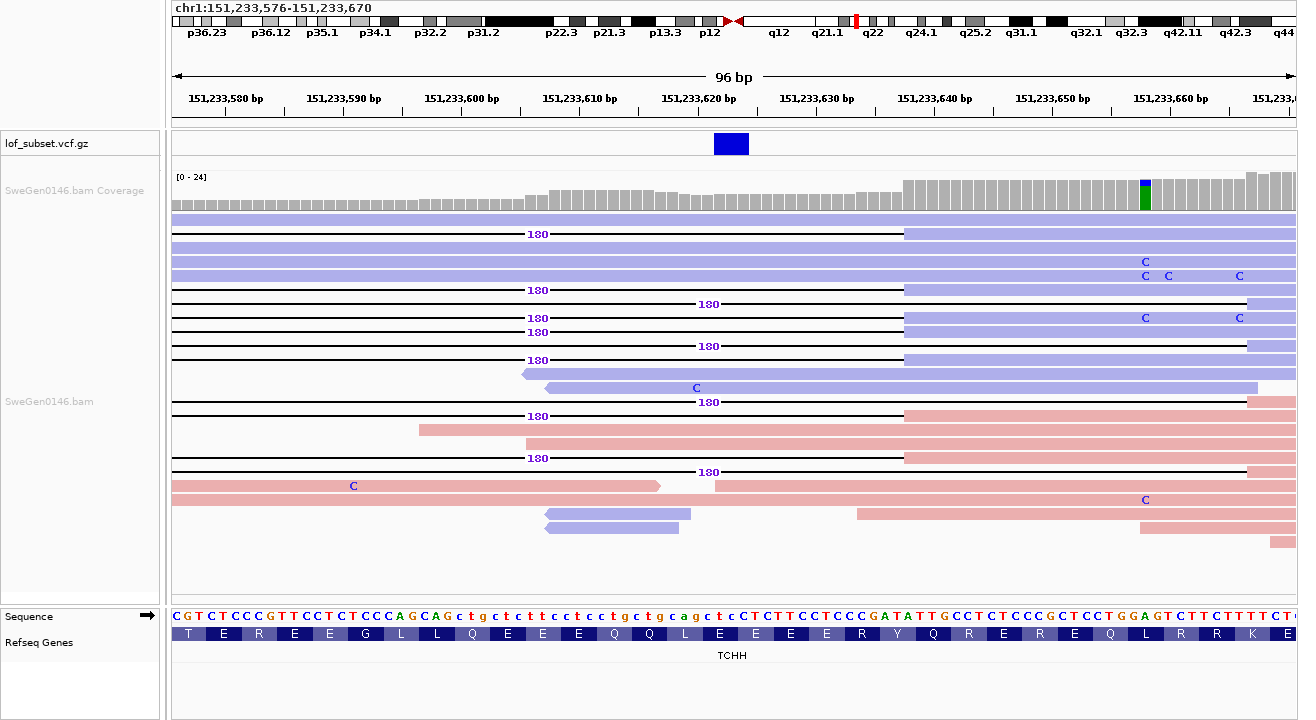

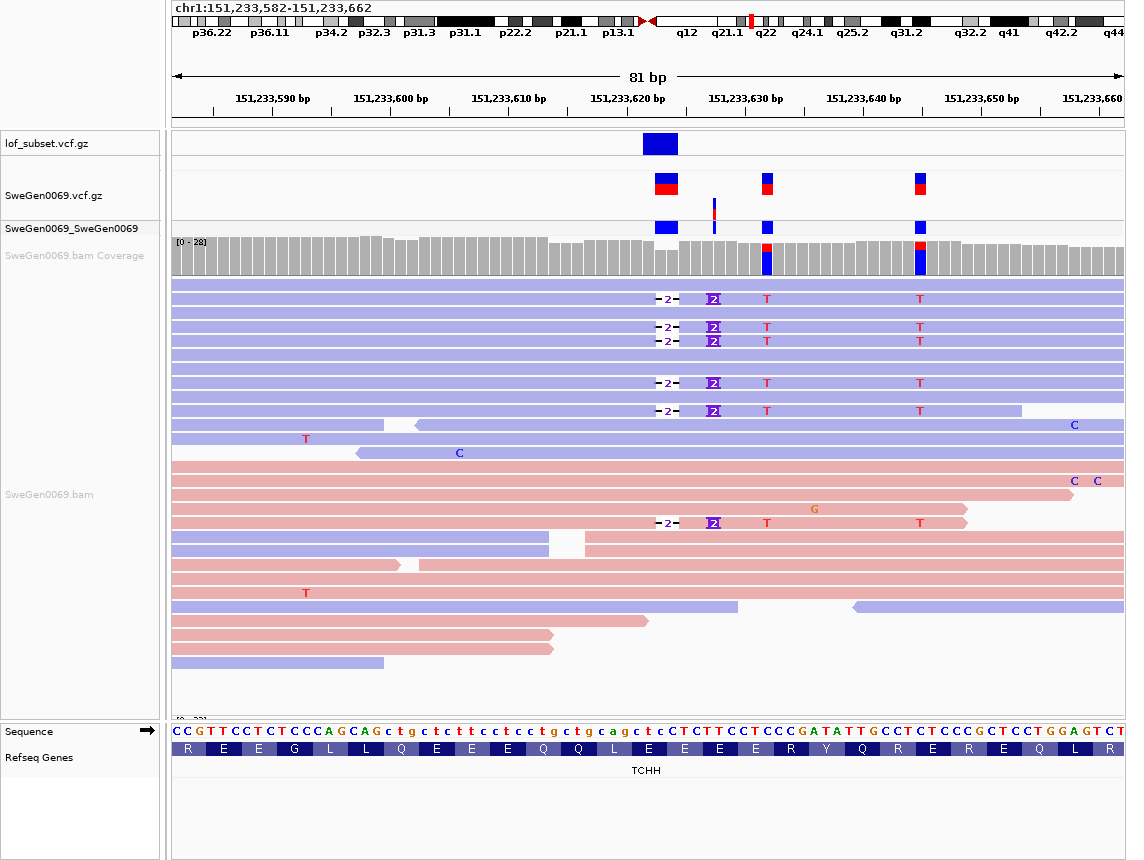


## Supplemental Figure S3c: Variant 3: chr2:161682863A>AT

Singleton variant. Well visible, no strand bias. Coverage 41, as expected.


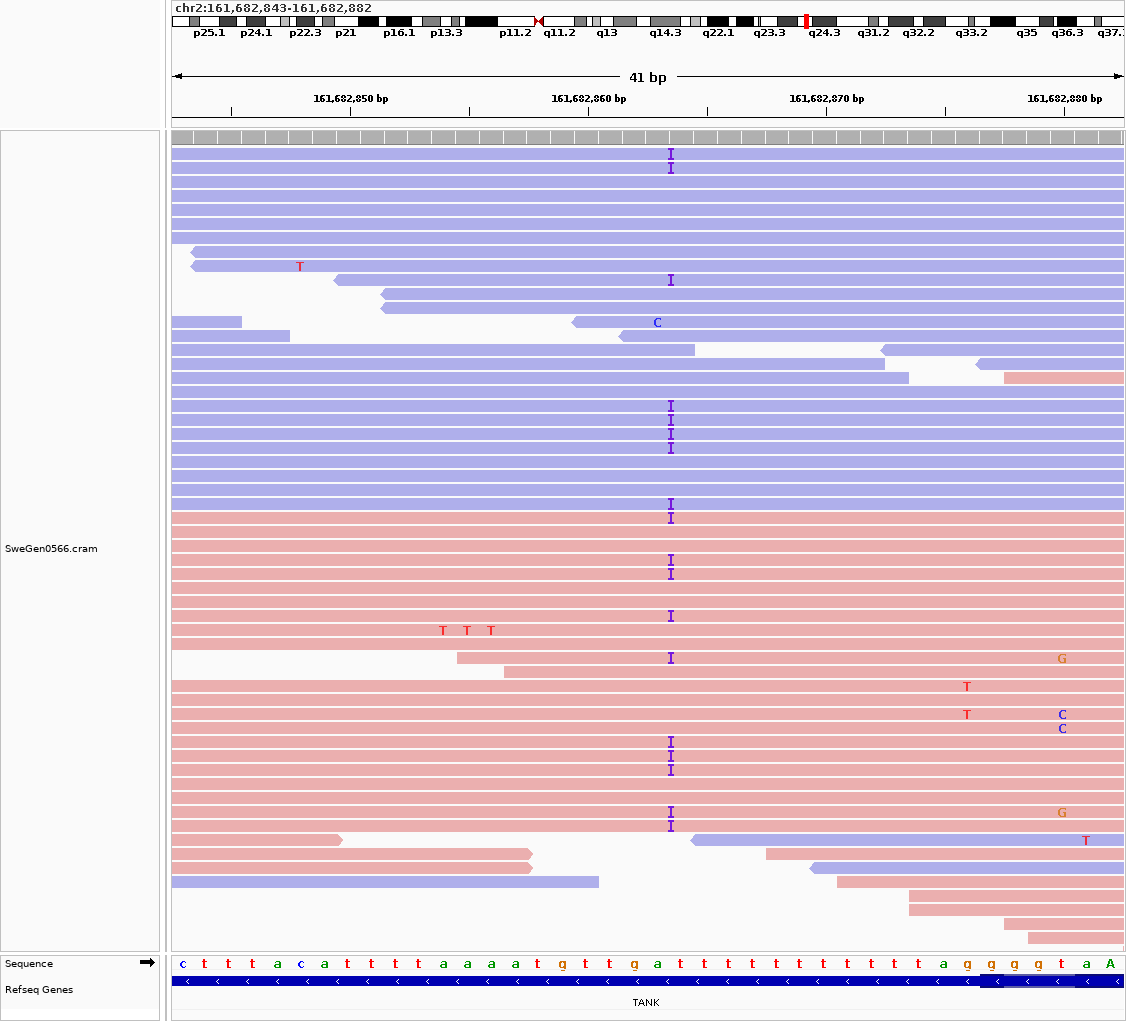


## Supplemental Figure S3d: Variant 4; chr3:38557623AGTGCCTG>*,A

Lower coverage in variant region and large fragments in original alignment (top). Visible in 926 in realigned BAM with coverage 25 (middle). Overlapping indel in 711 (coverage 19) and 282 (coverage 32) (bottom). Coverage around region normal and lower coverage expected in deletion. Deletion can be considered real but probably more complex than in the VCF


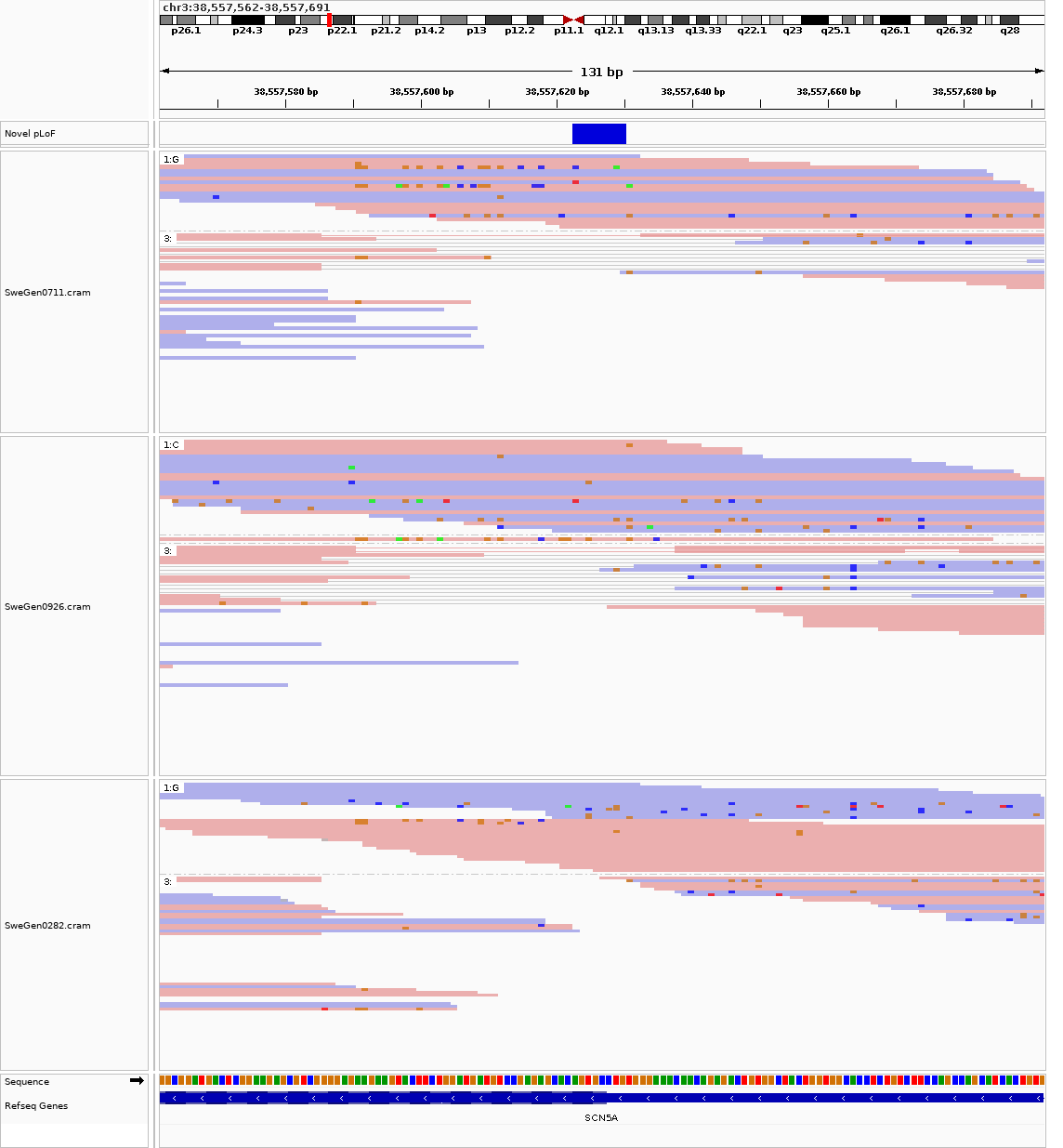


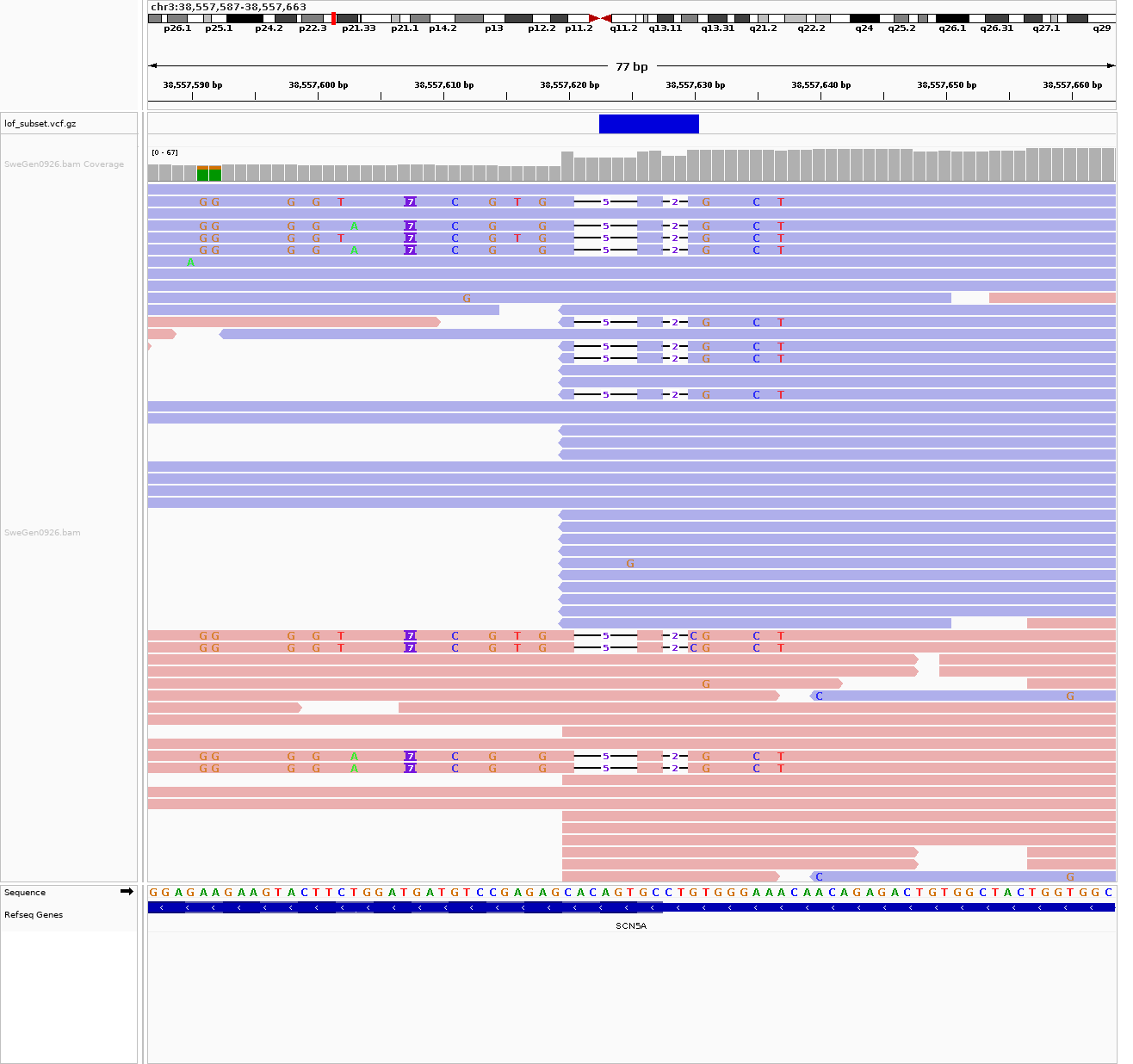

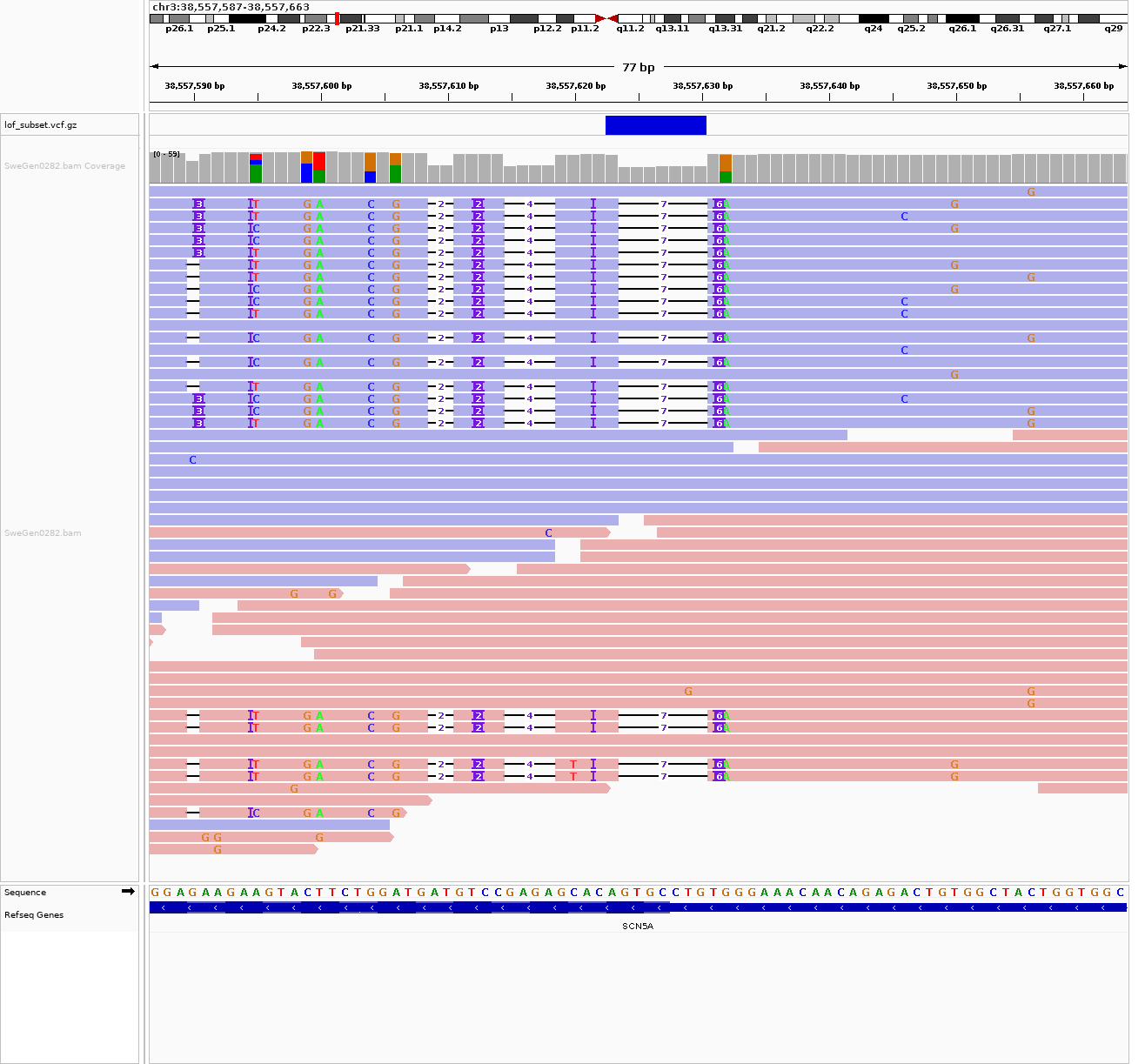


## Supplemental Figure S3e: Variant 5: chr3:47298950G>A

Called in 14 samples. Very well visible. Coverage ok (47). High probability that it is real.


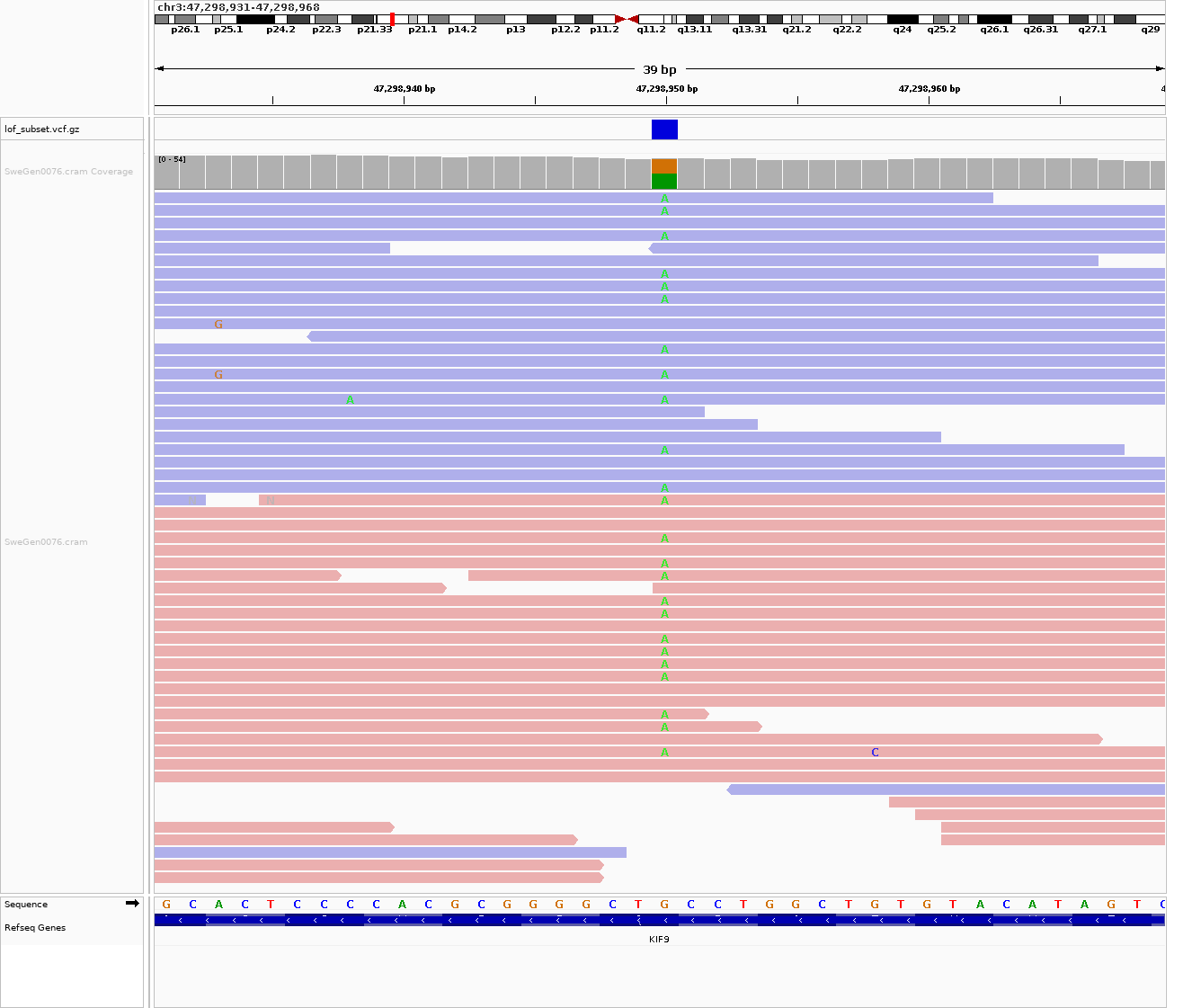


## Supplemental Figure S3f: Variant 6: chr6:10572556G>T

Not visible in original alignment. However, present in locally realigned BAM. No strand bias but coverage high (52). Only variant in this list not to pass all filters. Reads with variant share several indels. Probably artifact or misaligned reads.


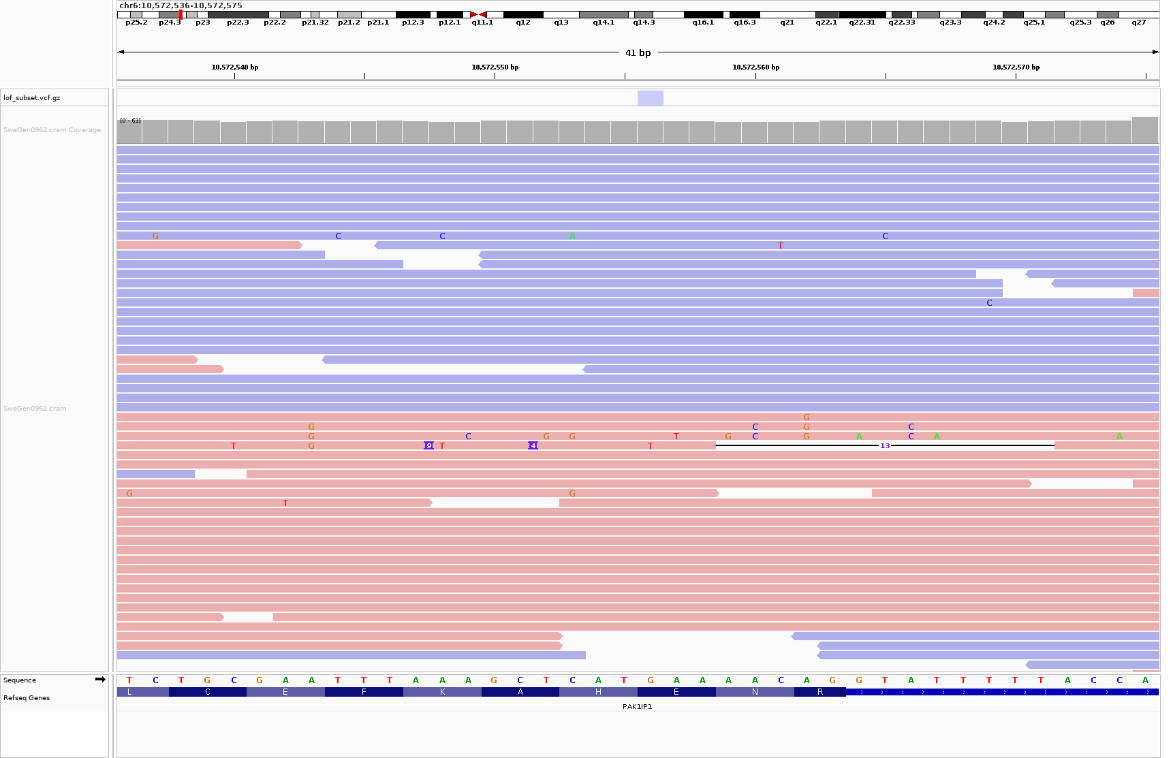


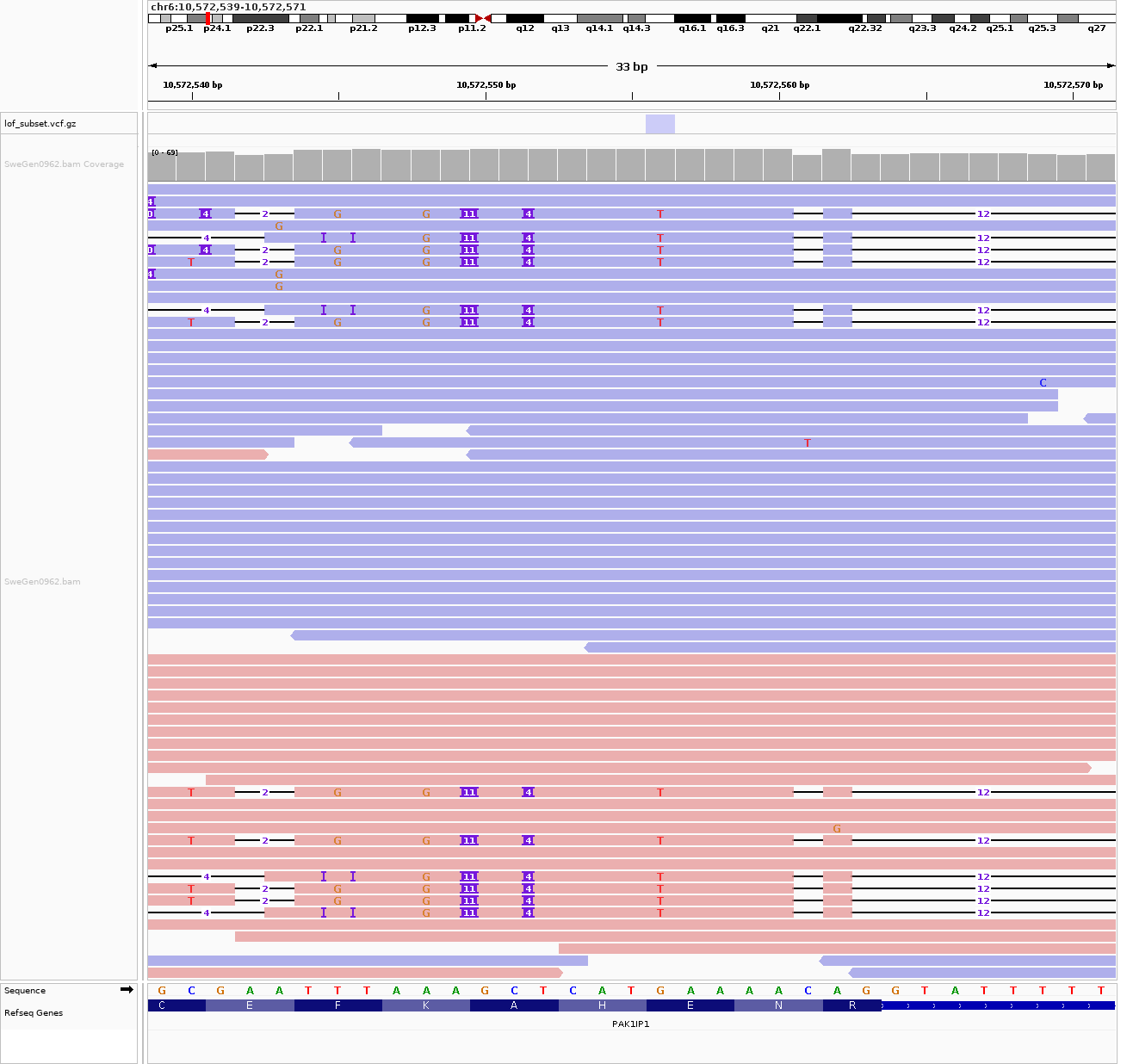


## Supplemental Figure S3g: Variant 7: chr9:82068921G>C

Visible in all three individuals where it was called. Coverage normal (29,39, 4¤). However, strand bias in 422 and 994. Is true but probably only in 986.


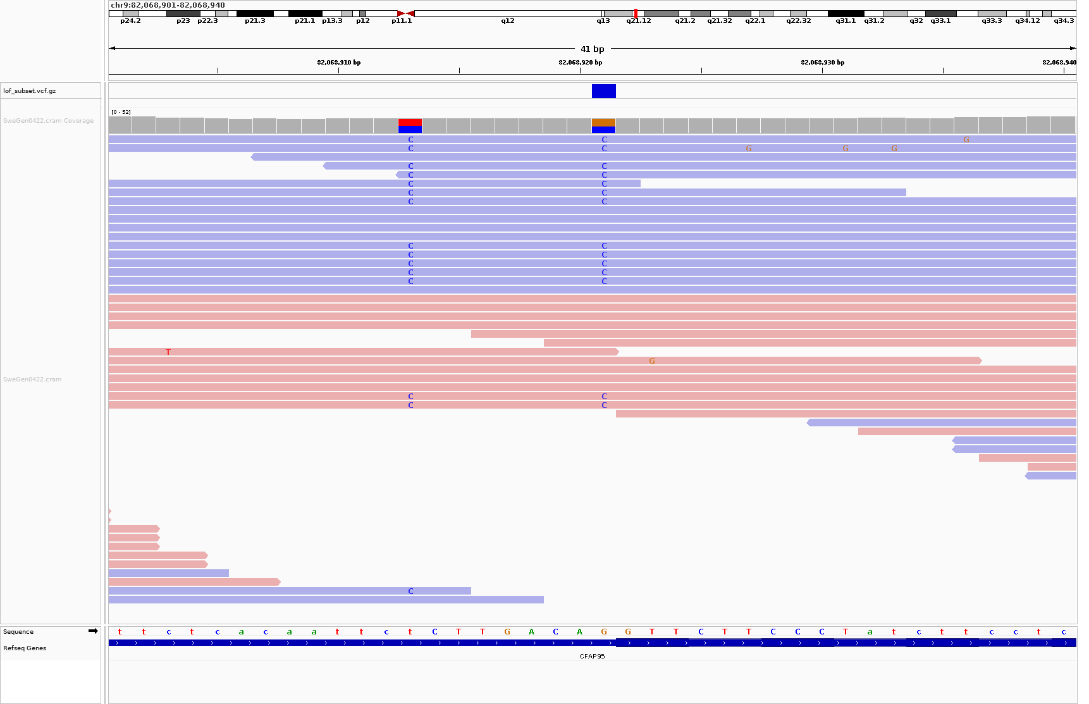


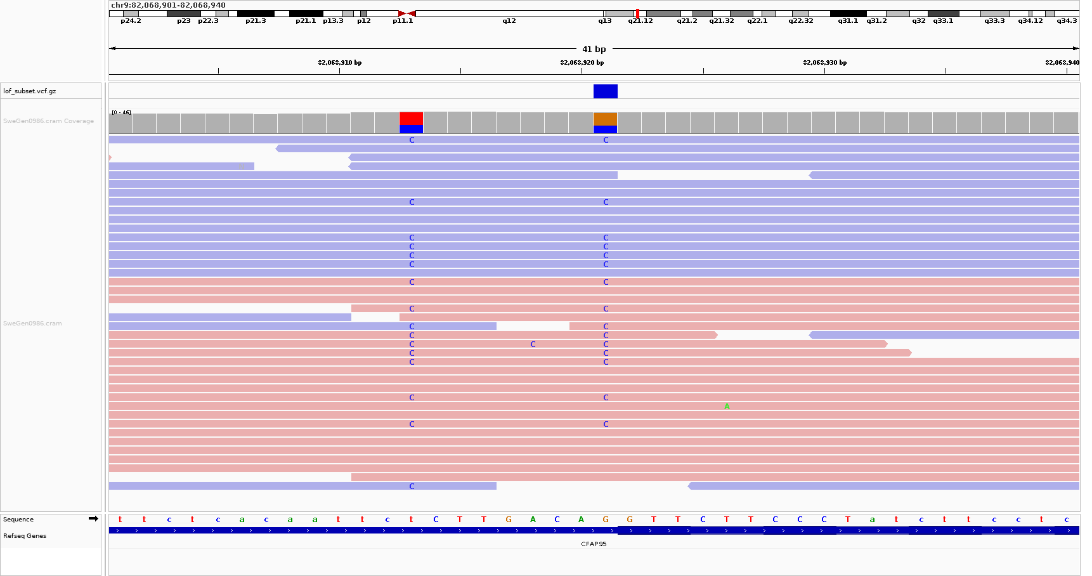

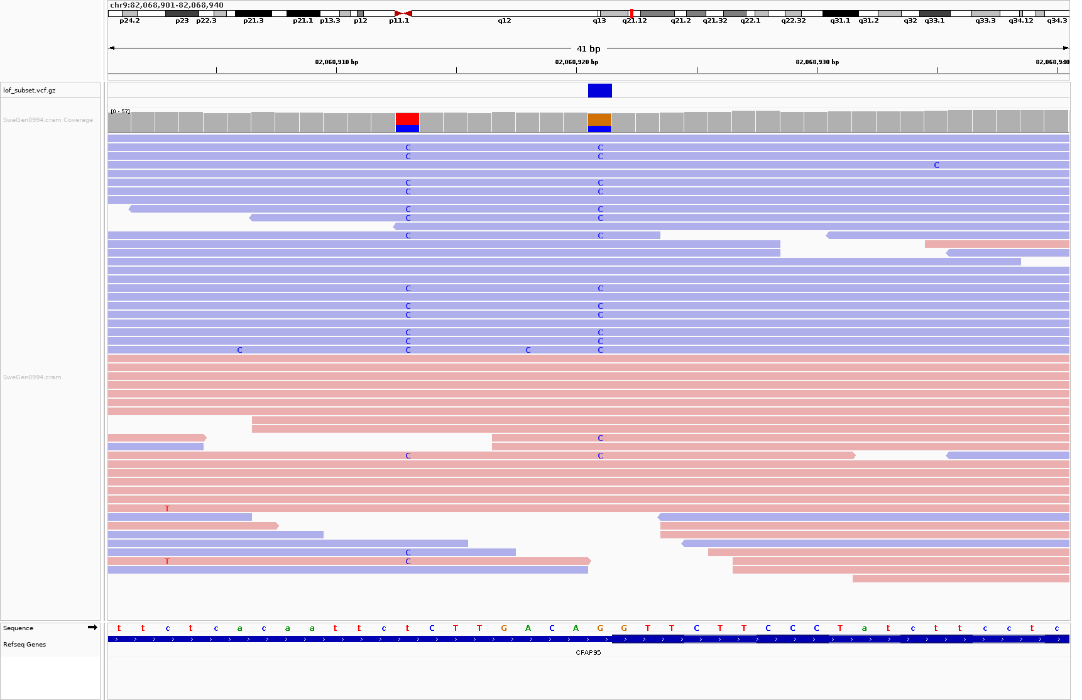


## Supplemental Figure S3h: Variant 8: chr11:117481727C>CG

Not visible in original alignments (example on top) but in realigned BAMs. Few supporting reads (max. 4), which also share several other indels. Probably false positive.


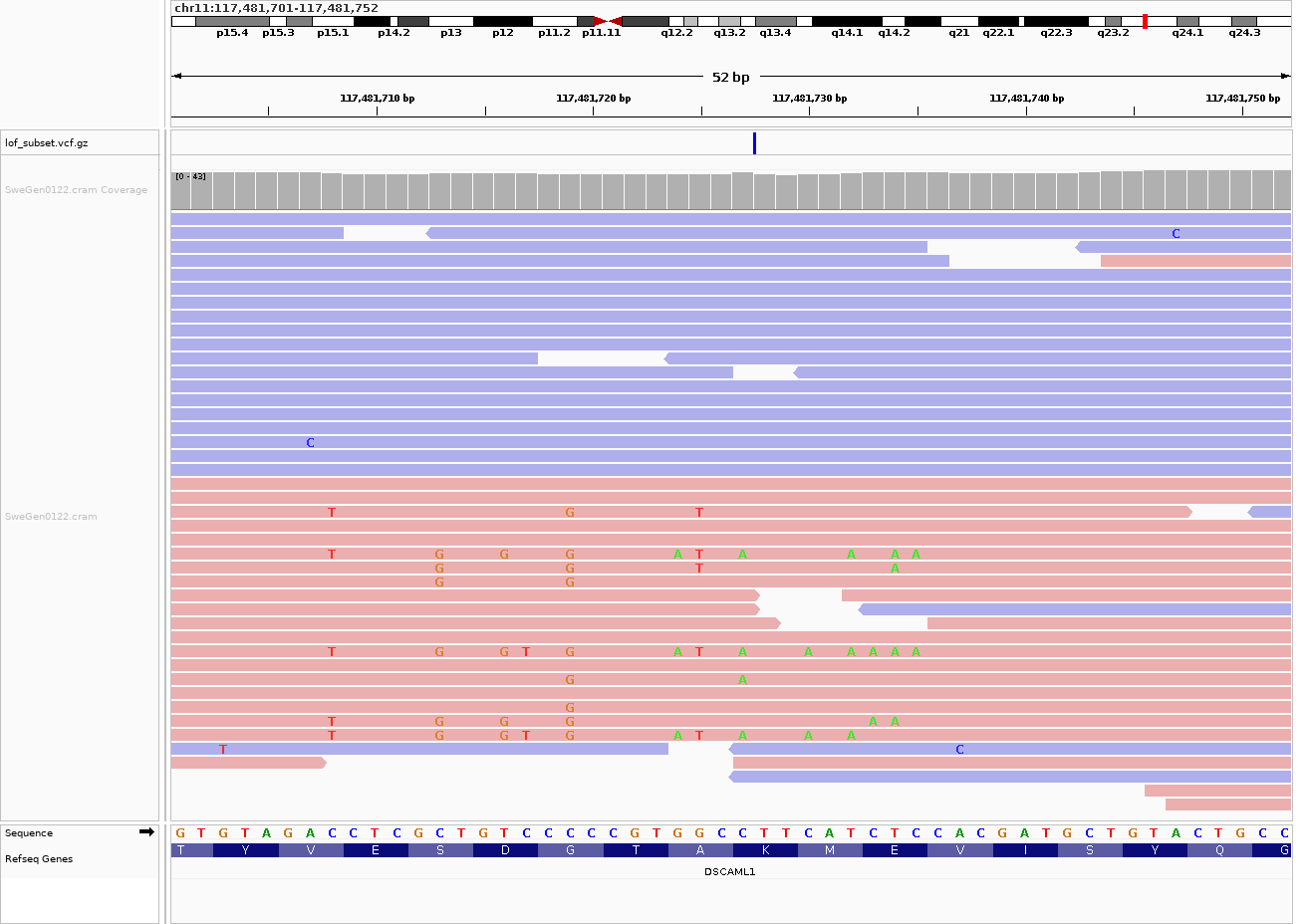


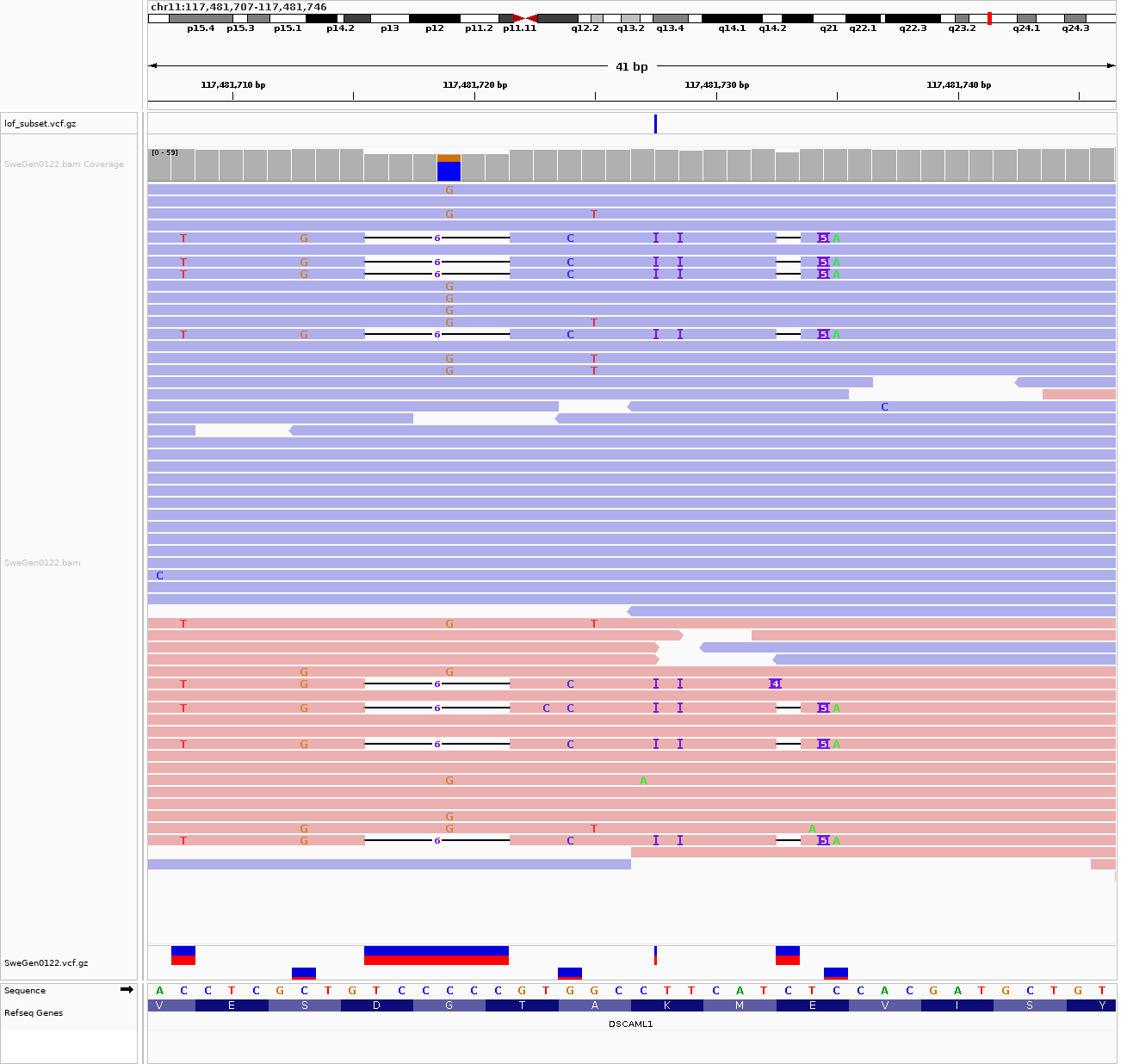


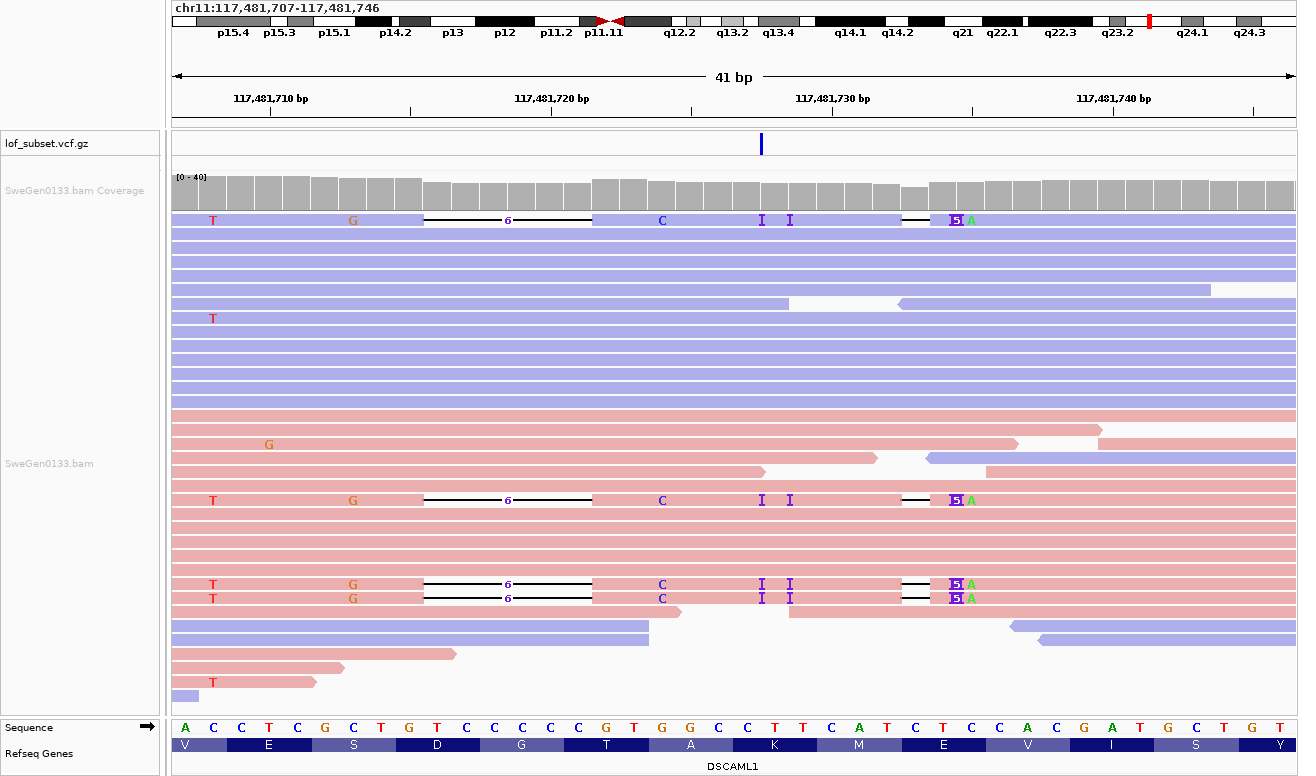


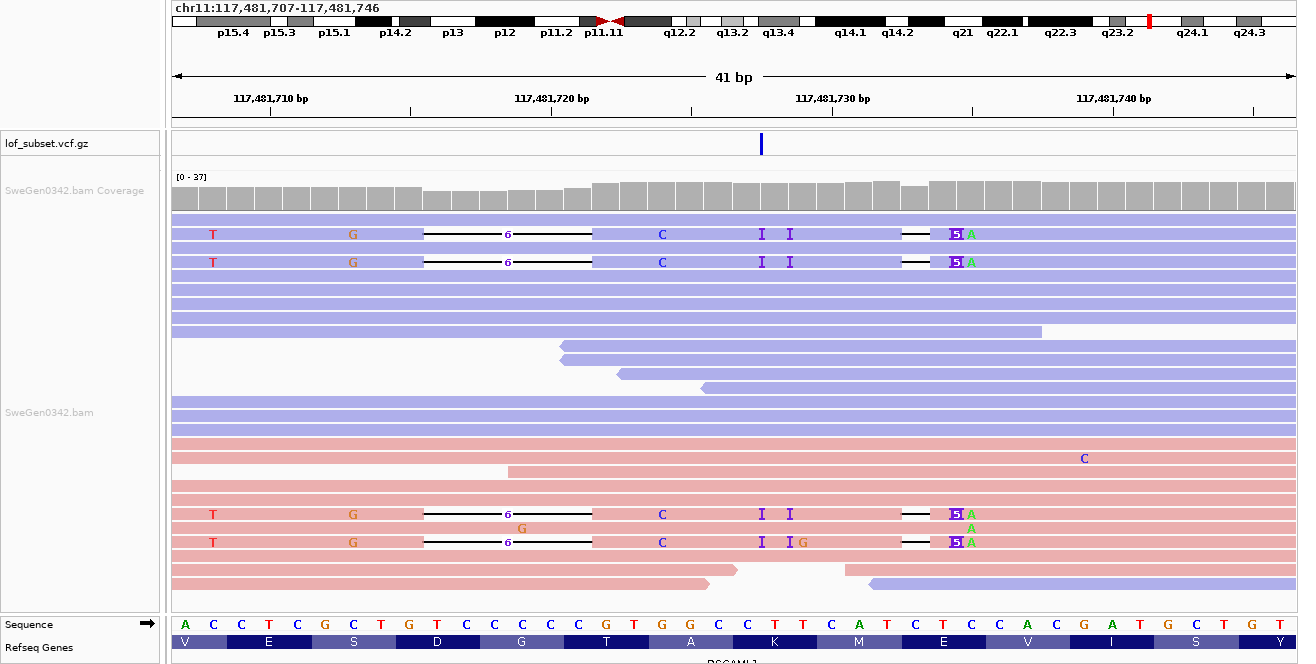


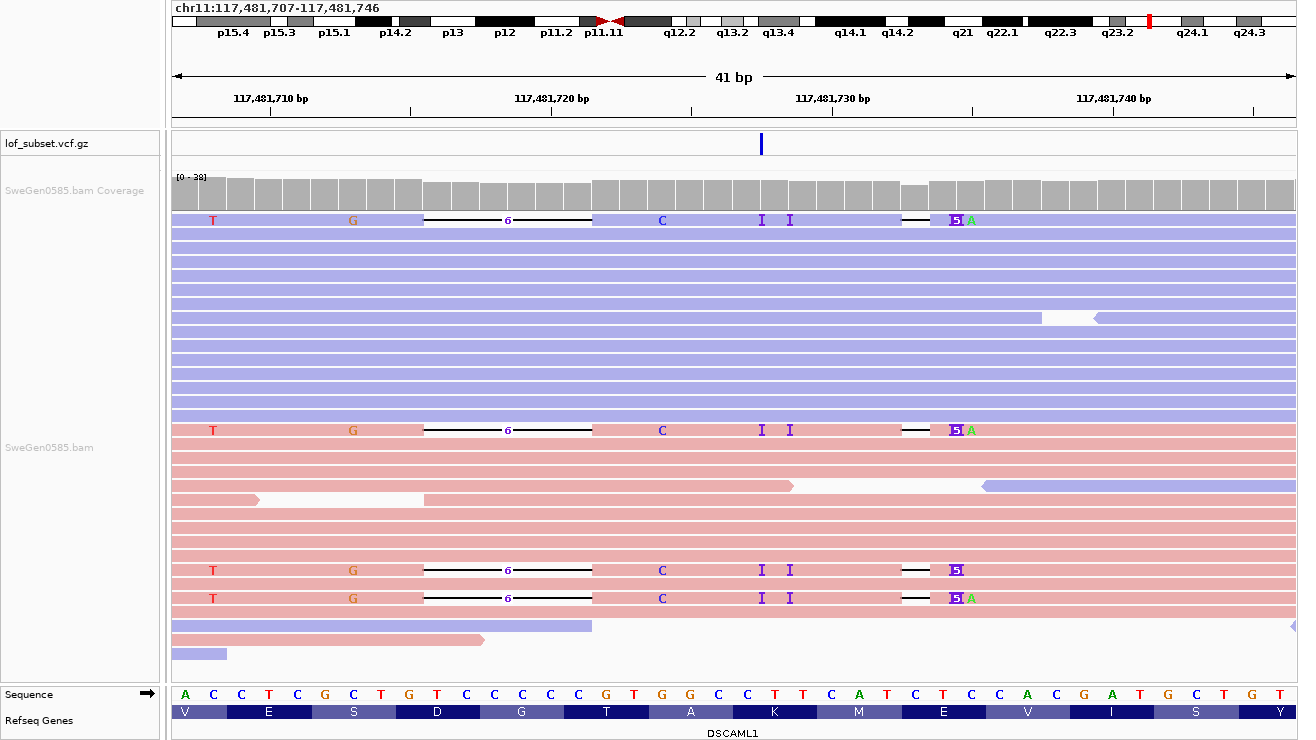


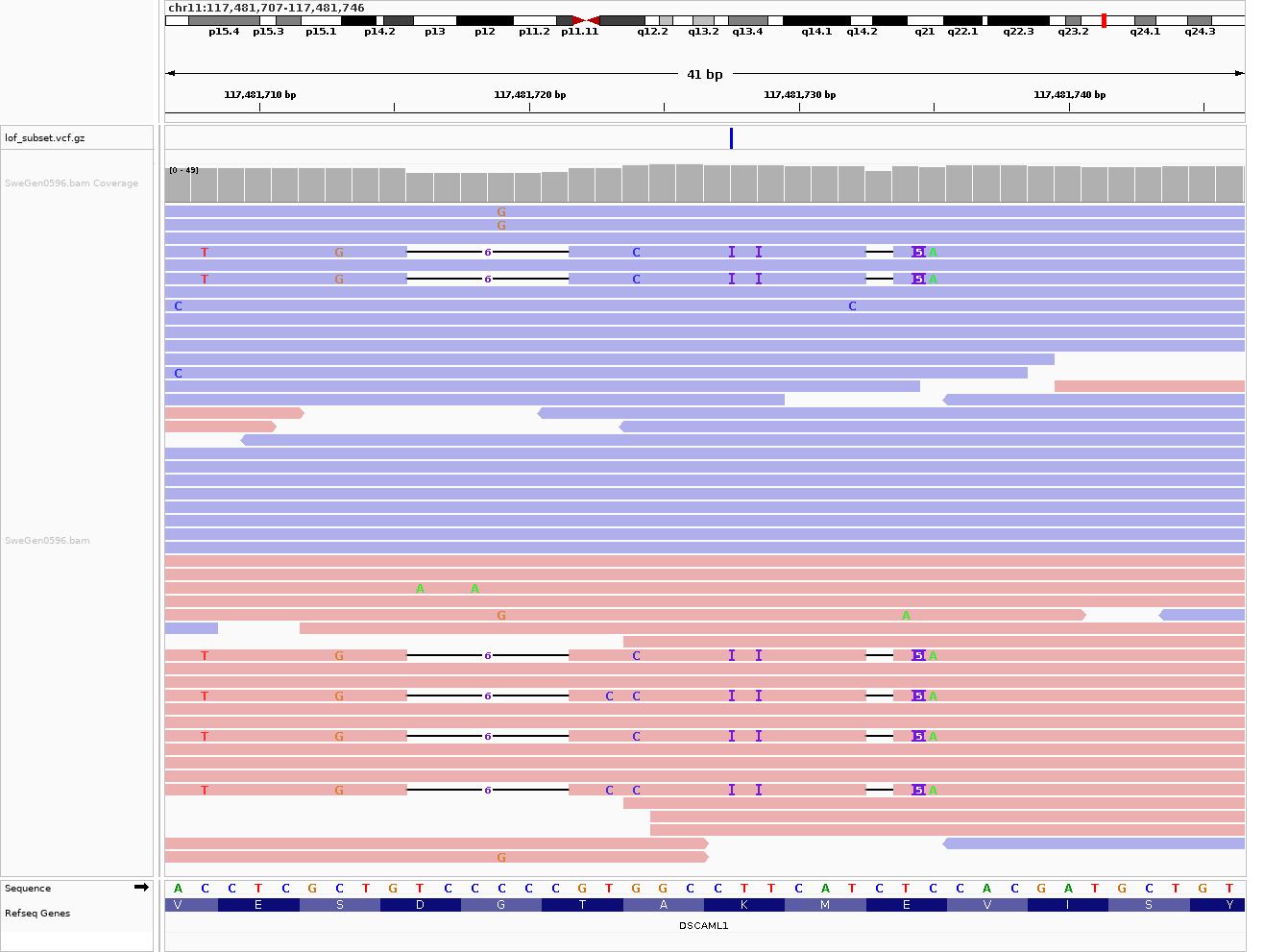


## Supplemental Figure S3i: Variant 9: chr12:50774778G>A

Visible in alignment but limited to one strand and at most four reads in each individual. Coverage normal. Might be artifact considering the polyA-sequences around the site.
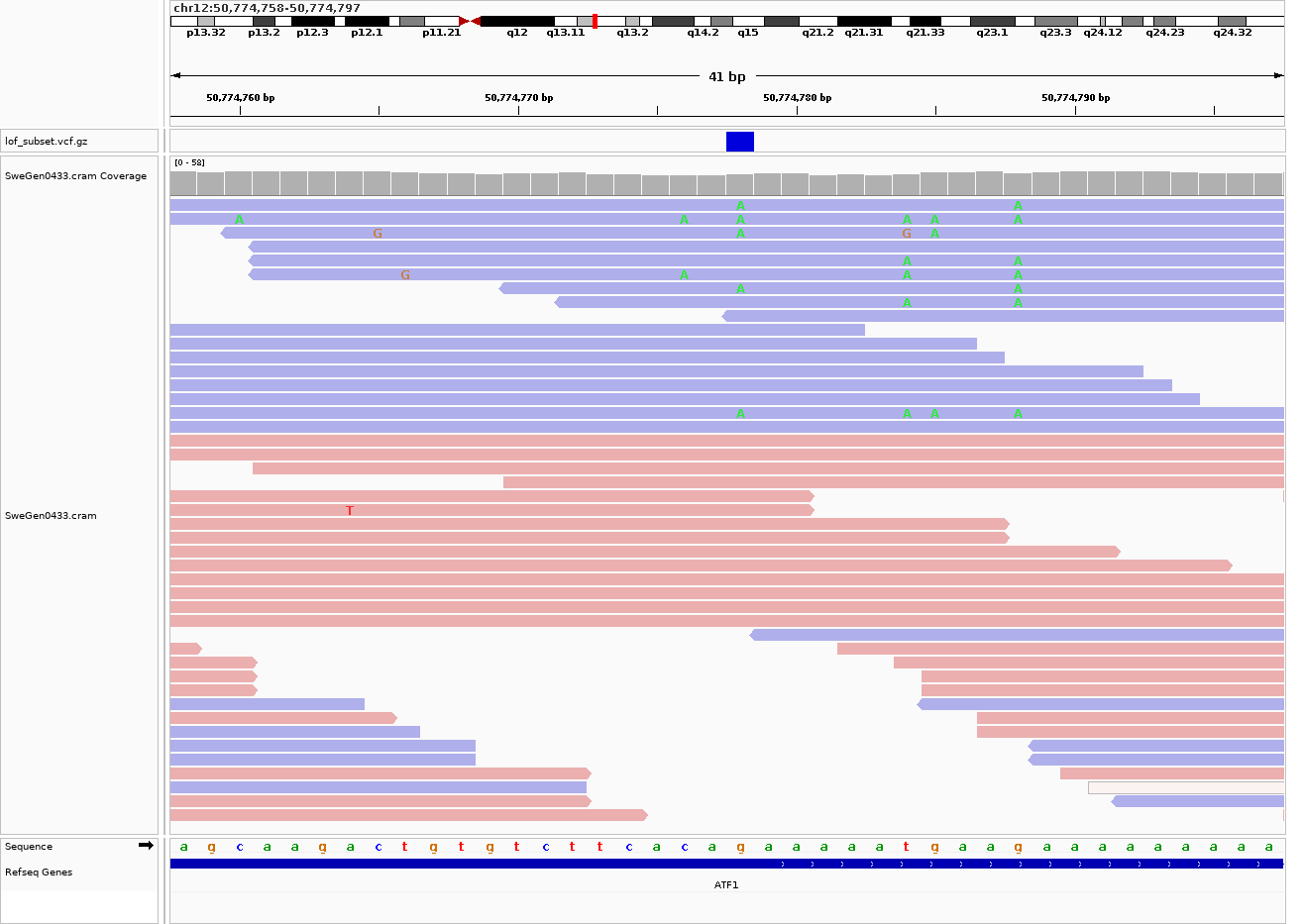

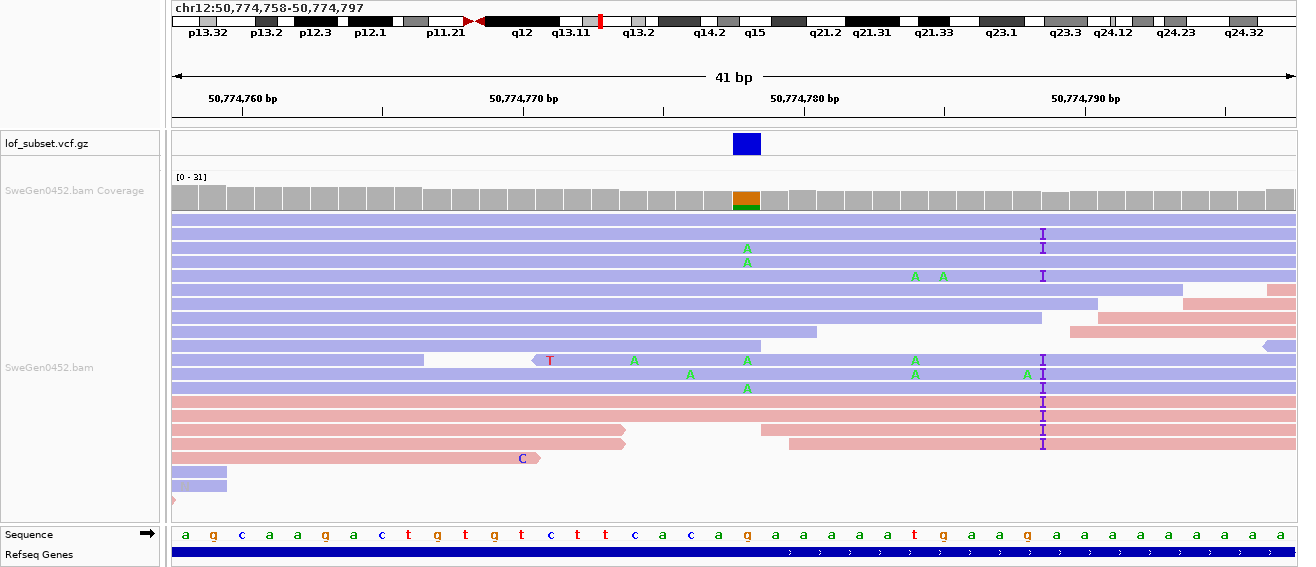


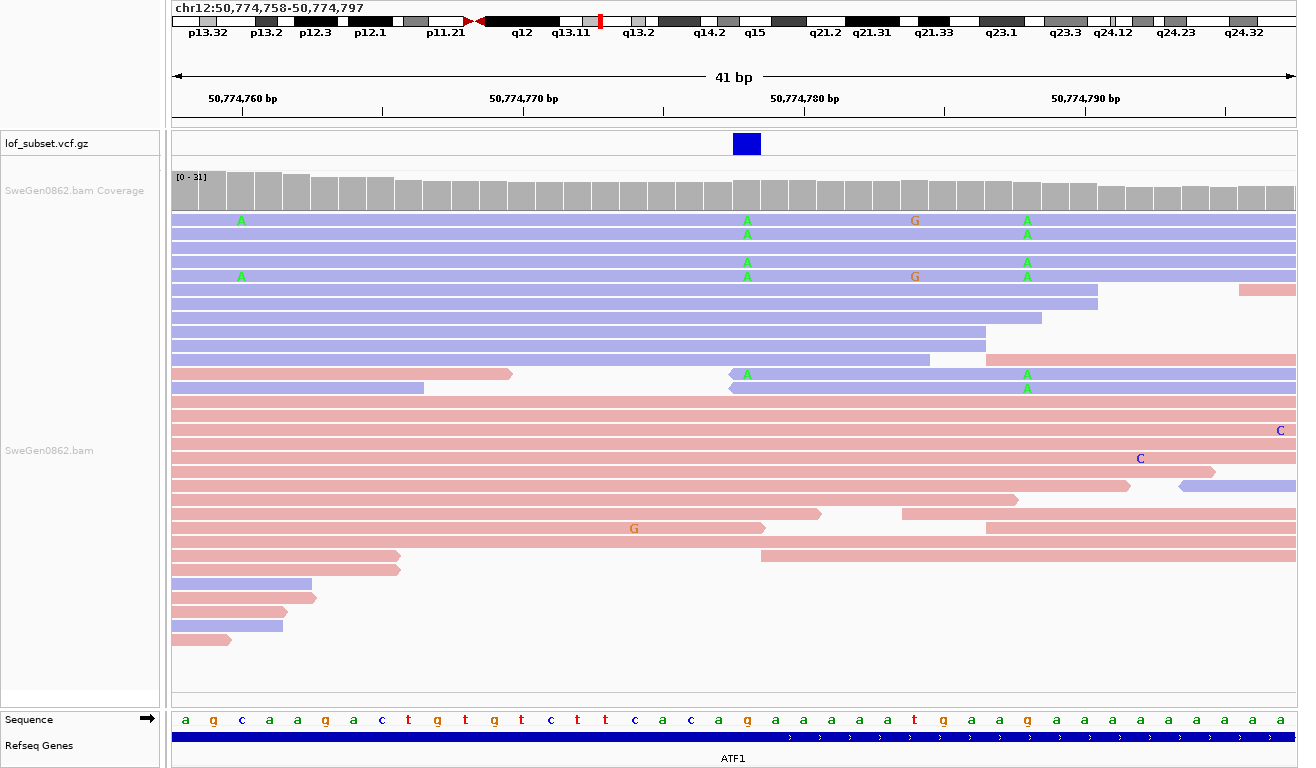


## Supplemental Figure S3j: Variant 10: chr19:58626502AGG>A

Not directly visible in alignment. Visible in realigned BAM but only on one strand. Therefore probably artifact.


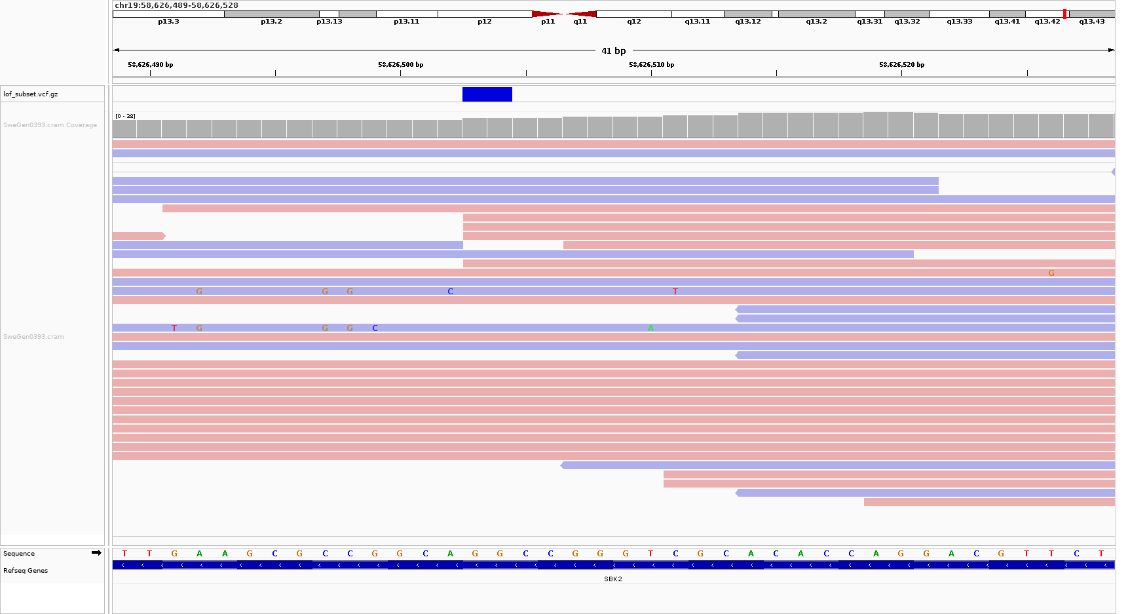


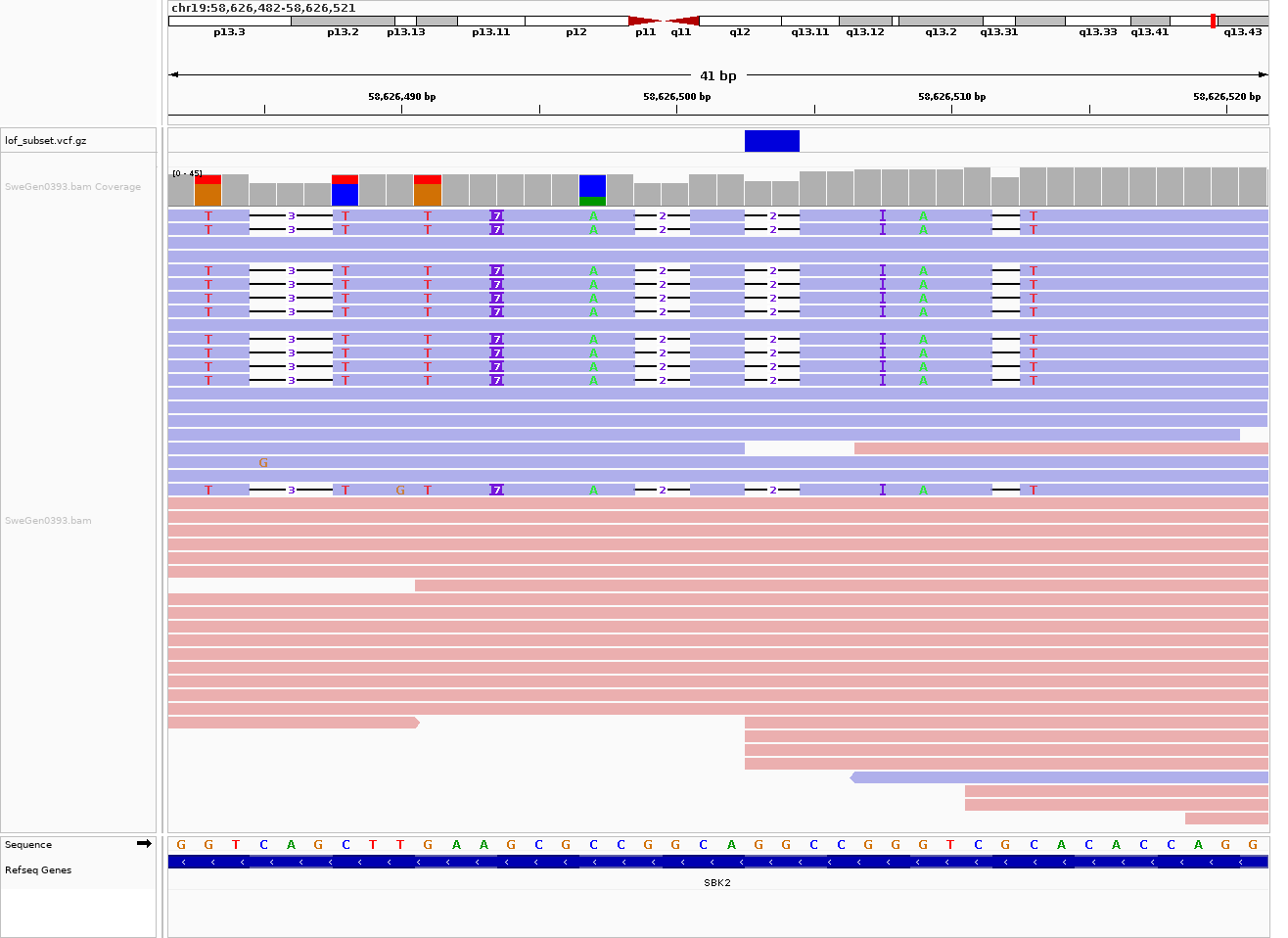


# Supplemental Figure S4

Comparison of the number of singletons per individual. Each dot represents one individual. The x-axis shows the number of singletons in the call set based on GRCh38, the y axis the singletons from the T2T-CHM13 call set. The dashed line has slope 1, i.e. individuals on this line had the same number of singleton calls using both references. All individuals had more singletons when using T2T-CHM13 and are therefore above this line.


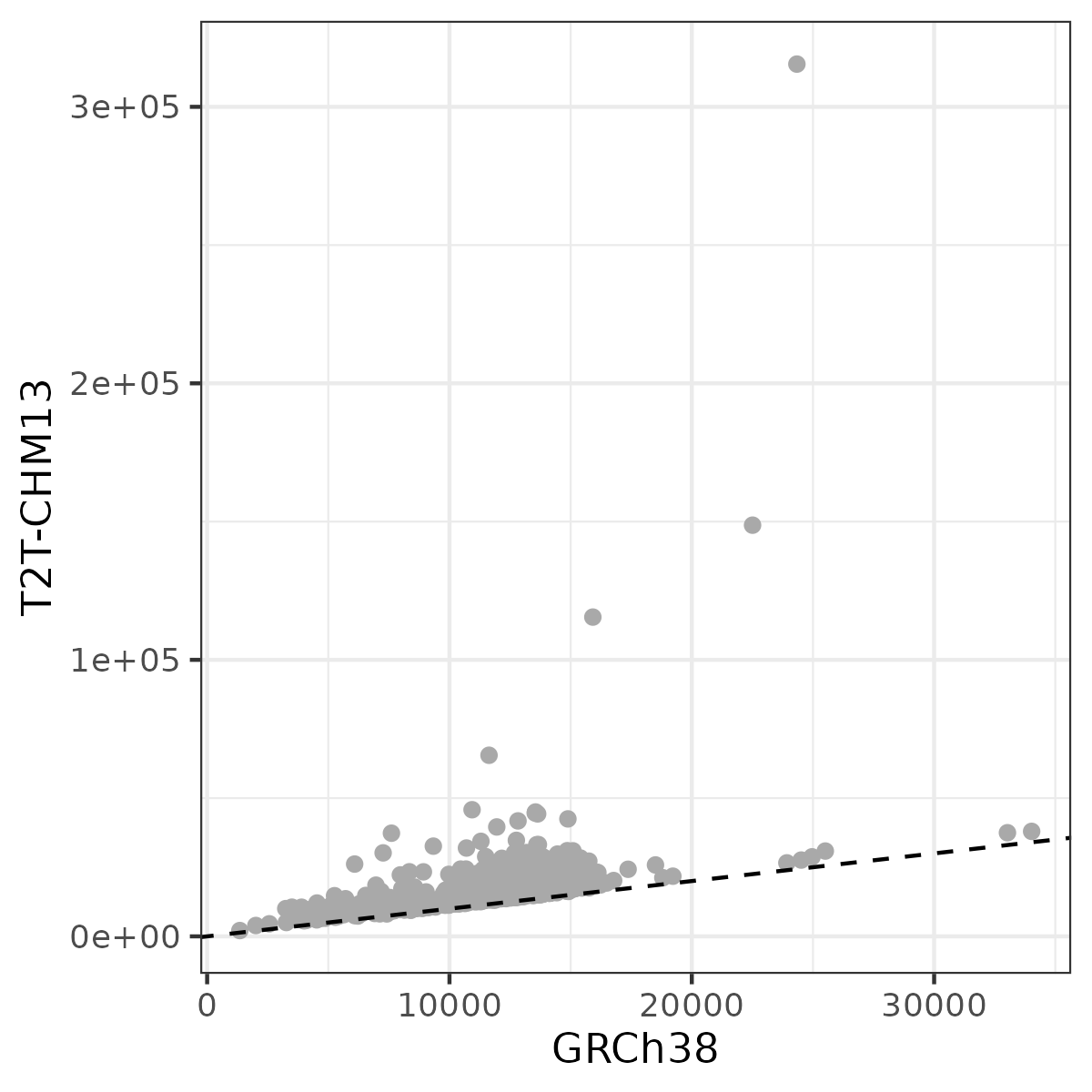


# Supplemental Figure S5

Summaries of variant characteristics in regions covered by the T2T Consortium-provided short-read accessibility mask. A) Comparison of overall variant counts, separated by variant type (SNV, indel or mixed, i.e., both) between variant calls from the different assemblies. B) Overall numbers of variants by predicted impact in the whole call set as determined by SnpEff for each assembly. Variants with impact rating MODIFIER were excluded. C) Distribution of allele frequencies in the three assemblies. D) Fraction of each variant type by allele frequency using T2T-CHM13. Frequencies were rounded to the closest multiple of 0.001 (1/1000).


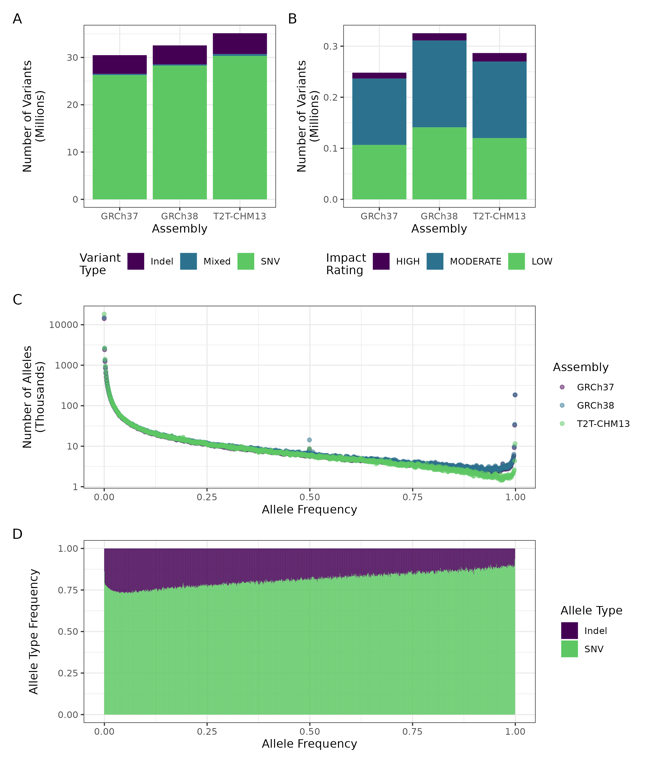

Supplement: Supplement 2 [file Supplemental_Figures.docx]
